# Amino Acid-Derived Metabolites from the Ascidian *Aplidium* sp.

**DOI:** 10.3390/md13063836

**Published:** 2015-06-16

**Authors:** Tae Hyung Won, Chang-Kwon Kim, So-Hyoung Lee, Boon Jo Rho, Sang Kook Lee, Dong-Chan Oh, Ki-Bong Oh, Jongheon Shin

**Affiliations:** 1 Natural Products Research Institute, College of Pharmacy, Seoul National University, San 56-1, Sillim, Gwanak, Seoul 151-742, Korea; E-Mails: wth123@snu.ac.kr (T.H.W.); kck1006@snu.ac.kr (C.-K.K.); sklee61@snu.ac.kr (S.K.L.); dongchanoh@snu.ac.kr (D.-C.O.); 2 Department of Agricultural Biotechnology, College of Agriculture and Life Science, Seoul National University, San 56-1, Sillim, Gwanak, Seoul 151-921, Korea; E-Mail: awhee84@naver.com; 3 Department of Biological Science, College of Life Science, Ewha Womans University, 52, Ewhayeodae-gil, Seodaemun, Seoul 120-750, Korea; E-Mail: nhm@ewha.ac.kr

**Keywords:** dipeptides, apliamides, *Aplidium* sp., Marfey’s analysis, bioactivities

## Abstract

Four new iodobenzene-containing dipeptides (**1**–**4**), a related bromotryptophan-containing dipeptide (**5**), and an iodophenethylamine (**6**) were isolated from the ascidian *Aplidium* sp. collected off the coast of Chuja-do, Korea. The structures of these novel compounds, designated as apliamides A–E (**1**–**5**) and apliamine A (**6**) were determined via combined spectroscopic analyses. The absolute configuration of the amino acid residue in **1** was determined by advanced Marfey’s analysis. Several of these compounds exhibited moderate cytotoxicity and significant inhibition against Na^+^/K^+^-ATPase (**4**).

## 1. Introduction

Ascidians (phylum Chordata, class Ascidiacea) are widely recognized as prolific sources of bioactive secondary metabolites that have attracted significant interest in the biomedical field [1,2,3,4,5,6,7]. The most noticeable example of an ascidian-derived drug is the recently developed anticancer agent, Yondelis (ecteinascidin 743), from the *Caribbean* ascidian *Ecteinascidia turbinata* [2,3,4,5,6,7,8,9]. Other notable examples under clinical trials for anticancer agents include aplidine from *Aplidium albicans* [10,11] and diazonamide from *Diazona angulata* [12].

The most distinctive feature of ascidian metabolites from other marine-derived compounds is the significant occurrence of amino-acid derived metabolites that have a great diversity of amino acid residues and functionalities [1]. Of these metabolites, those containing iodinated amino acid residues are scarce and have a limited distribution compared with other residues halogenated with chlorine or bromine [13]. Since the first isolation of two iodinated phenethylamines from *Didemnum* sp. [14], compounds of this structural class have been found from a few animals of the genera *Aplidium* [15] and *Didemnum* [16,17,18]*.* These metabolites have exhibited diverse bioactivities, such as cytotoxicity [14,15], antifungal activity [14] and the inhibition of glutathione reductase [15].

**Figure 1:**
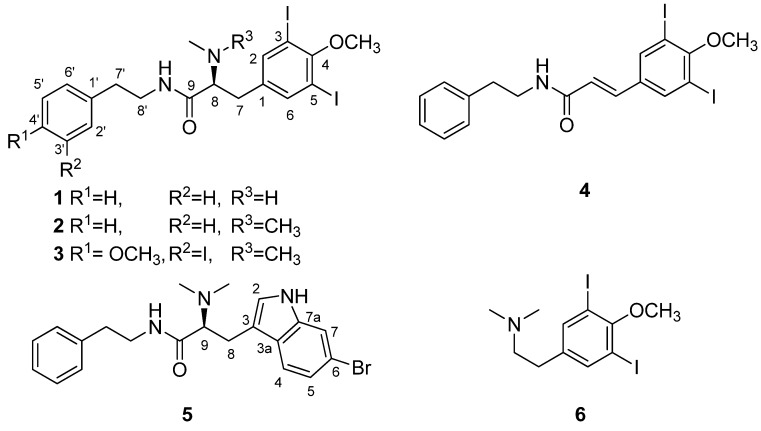
Structures of compounds **1**–**6**.

In our continuing search for bioactive metabolites from Korean ascidians [19,20,21,22], we recently encountered the reddish orange *Aplidium* sp. off the coast of Chuja-do, Korea, whose organic extract exhibited significant cytotoxicity (IC_50_ 38.6 µg/mL) for the A549 cancer cell-line. The bioassay-guided separation of the crude extract using diverse chromatographic methods yielded several peptide metabolites. In this study, we report the structural determination of six new compounds: apliamides A–D (**1**–**4**), four iodobenzene-containing dipeptides, apliamide E (**5**), a related bromotryptophan-containing dipeptide, and apliamine A (**6**), an iodinated phenethylamine (Figure 1). Several of these compounds exhibited moderate cytotoxicity for the K562 and A549 cell-lines, and apliamide D (**4**) significantly inhibited the action of Na^+^/K^+^-ATPase.

## 2. Results and Discussion

Compound **1** was isolated as an amorphous solid, which was analyzed by HRFABMS and determined to be C_19_H_22_N_2_O_2_I_2_, containing 9 degrees of unsaturation. However, the ^13^C NMR data showed only fifteen carbon signals. Of these signals, eight carbons in the downfield region of δ_C_ 142.0–91.1 showed highly disproportionate intensities. This spectroscopic feature, along with the corresponding proton signals at δ_H_ 7.66–7.11 in the ^1^H NMR data, strongly imply the presence of two symmetric benzene moieties (Table 1). A carbonyl carbon at δ_C_ 173.3 is indicative of an amide group, which was also supported by the characteristic absorption band at 1655 cm^−1^ in the IR data. The remaining signals in the ^13^C NMR data included one methine, three methylenes and two methyl carbons in the upfield region.

**Table 1:** NMR Data of Compounds **1** and **2** in MeOH-*d*_4_.

	1		2	
Position	δ_C_, Type	δ_H_, mult (*J* in Hz)	δ_C_, Type	δ_H_, mult (*J* in Hz)
1	138.2, C		139.8, C	
2/6	142.0, CH	7.66, s	142.0, CH	7.67, s
3/5	91.1, C		90.9, C	
4	159.5, C		159.1, C	
7	37.9, CH_2_	2.76, dd (13.5, 8.5)	34.9, CH_2_	2.78, dd (12.0, 5.0)
		2.82, dd (13.5, 6.5)		2.89, dd (12.0, 11.0)
8	66.0, CH	3.23, dd (8.5, 6.5)	71.8, CH	3.02, dd (11.0, 5.0)
9	173.3, C		172.2, C	
1′	140.2, C		140.3, C	
2′/6′	129.7, CH	7.11, d (8.0)	129.7, CH	7.05, d (7.5)
3′/5′	129.6, CH	7.24, t (8.0)	129.5, CH	7.22, t (7.5)
4′	127.4, CH	7.16, t (8.0)	127.3, CH	7.14, t (7.5)
7′	36.7, CH_2_	2.57, ddd (14.0, 7.5, 6.5)	36.8, CH_2_	2.51, ddd (14.0, 7.5, 7.0)
		2.67, ddd (14.0, 8.0, 7.5)		2.64, ddd (14.0, 7.5, 7.0)
8′	41.7, CH_2_	3.25, ddd (14.0, 7.5, 7.5)	41.6, CH_2_	3.17, ddd (13.5, 7.0, 7.0)
		3.44, ddd (14.0, 8.0, 6.5)		3.43, ddd (13.5, 7.5, 7.5)
4-OMe	61.1, CH_3_	3.77, s	61.1, CH_3_	3.76, s
8-NMe	34.0, CH_3_	2.28, s	42.5, CH_3_ (2C)	2.29, s (6H)

**Figure 2:**
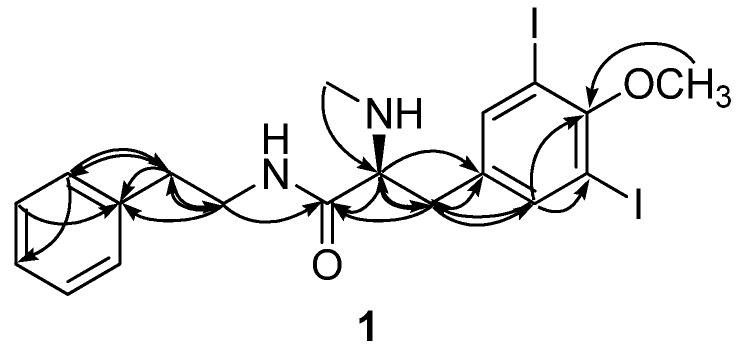
Selected HMBC correlations for compound **1**.

The structure of **1** was then elucidated by a combination of ^1^H–^1^H COSY, HSQC and HMBC analyses. All of the protons and their attached carbons were precisely matched by the HSQC data. The long-range couplings of two singlet methine protons at δ_H_ 7.66 with the neighboring carbons in the HMBC data revealed the presence of a 1,3,4,5-tetrasubstituted benzene moiety (Figure 2). The unusually strong shielding of the C-3 and C-5 carbons at δ_C_ 91.1 indicated the placement of iodine atoms at these positions. Similarly, a methoxy group was located at C-4 according to the deshielding of this carbon at δ_C_ 159.5 as well as its long-range correlation with the methoxy proton at δ_H_ 3.77. Thus, the 3,5-diiodo-4-methoxyphenyl moiety (C-1~C-6 and 4-OMe) was adequately determined. The sequential linkage at C-1 with a methylene (C-7, δ_C_ 37.9, δ_H_ 2.76 and 2.82), a methine (C-8, δ_C_ 66.0, δ_H_ 3.23) and a carbonyl carbon (C-9, δ_C_ 173.3) was verified by the COSY correlations between H-7 and H-8, as well as several HMBC correlations among these protons and the neighboring carbons, as follows: H-2, -6/C-7; H-7/C-1, C-2, C-6, C-8, and C-9; H-8/C-1, C-7, and C-9. An *N*-methyl group (δ_C_ 34.0, δ_H_ 2.28) was located at C-8 according to the mutual HMBC correlations with the C-8 methine, H-8/C-NMe; H-NMe/C-8.

The proton-proton coupling patterns among the five protons at δ_H_ 7.24–7.11 in the COSY data indicated the presence of a phenyl group (C-1′–C-6′), which was confirmed by the HMBC correlations between these aromatic protons and the neighboring carbons. The sequential linkage of this phenyl group with two methylenes (C-7′, δ_C_ 36.7, δ_H_ 2.57 and 2.67; C-8′, δ_C_ 41.7, δ_H_ 3.25 and 3.44) was also confirmed by the COSY correlations among the methylene protons; this confirmation was aided by the HMBC data of H-2′, -6′/C-7′; H-7′/C-1′, C-2′, C-6′ and C-8′; H-8′/C-1′. The carbon and proton chemical shifts of the C-8′ methylene group suggested the direct attachment of a nitrogen at this position. Thus, the phenethylamine moiety was determined.

The connectivity of the two phenyl-containing partial structures was also determined by the HMBC data. The long-range couplings of the H-8′ methylene protons at δ_H_ 3.25 and 3.44 with the C-9 carbonyl carbon at δ_C_ 173.3 suggested the presence of an amide linkage. This interpretation was further supported by the ESI-Q-TOF-MS/MS analysis in which a noticeable fragment resulting from the α-cleavage of an amide bond was obtained (See Supporting Information, Figure S1). Thus, the structure of **1**, designated apliamide A, was determined to be a new iodobenzene-containing dipeptide.

Apliamide A (**1**) possessed an asymmetric carbon center at the C-8 of the iodotyrosine-derived unit. The absolute configuration of this amino acid residue was determined by advanced Marfey’s analysis. [23,24] After the acidic hydrolysis of **1**, the ESI-LC/MS analysis of the hydrolysate adducts with l- and d-FDAA (Marfey’s reagent, 1-fluoro-2-4-dinitrophenyl-5-l(or d)-alanine amide) confirmed the NMR-based structural elucidation of the iodotyrosine-derived unit. An L configuration was also assigned at C-8 from the shorter LC retention time of the l-FDAA adduct compared with the countering d-FDAA adduct (See Experimental Section). The elution order of l- and d-FDAA adduct was dependent on the difference in hydrophobicity between the α-carboxyl group and the iodinated phenyl group of an amino acid residue. An l-FDAA adduct with a trans-type arrangement of two more hydrophobic substituents at both α-carbons of an amino acid and l-alanine amide moieties is eluted before d-FDAA adduct with a cis-type arrangement of those [25].

The molecular formula of apliamide B (**2**) was established as C_20_H_25_N_2_O_2_I_2_ via HRFABMS analysis. The ^1^H and ^13^C NMR data of this compound were very similar to those of **1** with the additional presence of signals representing a methyl group (δ_C_ 42.5, δ_H_ 2.29), which was the most noticeable difference (Table 1). The combined 2D NMR data were nearly identical to those of **1**, implying the same aromatic dipeptide nature. The new methyl group was located at 8-NMe based on its carbon and proton chemical shifts, singlet proton multiplicity, and the crucial HMBC correlations at H-8/C-NMe and H-NMe/C-8. This interpretation was confirmed by ESI-MS/MS analysis in which a fragment containing the 8-*N*,*N*-dimethyl group was obtained (See Supporting Information, Figure S1). The similar specific rotation of +11.2 and +12.2 for **1** and **2**, respectively, suggested the same L configuration at C-8 for **2**. Thus, the structure of apliamide B (**2**) was determined as the 8-NMe derivative of **1**.

Apliamide C (**3**) was isolated as an amorphous solid with a molecular formula of C_21_H_25_N_2_O_3_I_3_ via HRFABMS analysis. The ^13^C and ^1^H NMR data of this compound were reminiscent of **2**, revealing the same dipeptide features. However, the detailed examination of the NMR data revealed that the phenyl moiety (C-1′-C-6′) was substituted by the following two substituents: an iodine and a methoxy group (δ_C_ 56.9, δ_H_ 3.82) (Table 2). The chemical shifts and coupling patterns of the three aromatic protons at δ_H_ 6.82 (1 H, d, *J* = 8.0 Hz), 6.97 (1 H, dd, *J* = 8.0, 2.0 Hz), and 7.51 (1 H, d, *J* = 2.0 Hz) in the ^1^H NMR data were characteristic of an ABX spin system. Based on the results of the combined 2D NMR data, including the key HMBC correlations of these protons with neighboring carbons, the iodine and methoxy groups were located at C-3′ and C-4′, respectively. The ESI-MS/MS data provided a fragment derived by the α-cleavage of the amide bond, supporting the NMR-determined structure (See Supporting Information, Figure S1). Thus, the structure of apliamide C (**3**) was determined as a dipeptide containing two iodinated phenyl moieties.

**Table 2:** NMR Data of Compounds **3** in MeOH-*d*_4_ and **4** in CDCl_3_.

	3		4	
Position	δ_C_, Type	δ_H_, mult (*J* in Hz)	δ_C_, Type	δ_H_, mult (*J* in Hz)
1	135.4, C		134.8, C	
2/6	142.3, CH	7.72, s	138.9, CH	7.86, s
3/5	91.5, C		90.8, C	
4	160.2, C		159.8, C	
7	33.9, CH_2_	2.94, dd (11.5, 11.5)	138.7, CH	7.41, d (15.0)
		3.27, dd (11.5, 5.0)		
8	70.8, CH	3.73, dd (11.5, 5.0)	122.1, CH	6.21, d (15.0)
9	167.1, C		165.0, C	
1′	133.9, C		137.3, C	
2′	131.0, CH	7.51, d (2.0)	128.8, CH	7.22, d (7.5)
3′	86.5, C		128.7, CH	7.33, t (7.5)
4′	158.5, C		126.7, CH	7.25, d (7.5)
5′	112.2, CH	6.82, d (8.0)	128.7, CH	7.33, d (7.5)
6′	140.6, CH	6.97, dd (8.0, 2.0)	128.8, CH	7.22, d (7.5)
7′	34.9, CH_2_	2.36, ddd (14.0, 7.5, 7.5)	35.6, CH_2_	2.89, t (7.0)
		2.53, ddd (14.0, 7.5, 5.5)		
8′	41.7, CH_2_	3.22, ddd (13.5, 7.5, 5.5)	40.8, CH_2_	3.67, dt (7.0, 6.5)
		3.34, ddd (13.5, 7.5, 7.5)		
4-OMe	61.2, CH_3_	3.79, s	60.8, CH_3_	3.86, s
4′-OMe	56.9, CH_3_	3.82, s		
8-NMe	42.5, CH_3_ (2C)	3.87, s (6H)		
8′-NH		ND		5.57, t (6.5)

**Figure 3:**
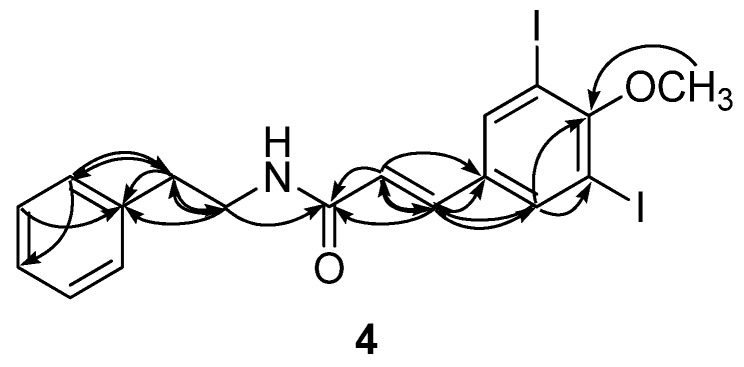
Selected HMBC correlations for compound **4**.

The molecular formula of apliamide D (**4**) was deduced as C_18_H_17_NO_2_I_2_ via HRFABMS analysis. The ^13^C and ^1^H NMR data of this compound were similar to those of **1**, revealing the same dipeptide nature for **4**. The most noticeable differences in the NMR data were the lack of signals for the 8-NMe group, consistent with the mass spectral data. In addition, the C-7 and C-8 aliphatic carbons were replaced with olefinic methines (δ_C_ 138.7 and 122.1, δ_H_ 7.41 and 6.21) in **4** (Table 2). These interpretations were confirmed by the combined 2D NMR data. Crucial evidence was provided by the HMBC data in which several correlations were obtained between the olefinic protons and the neighboring carbons, as follows: H-7 (δ_H_ 7.41)/C-1, C-2, C-6, C-8, and C-9; H-8 (δ_H_ 6.21)/C-1, C-7, and C-9 (Figure 3). The *E* configuration was assigned to the C-7 double bond based on the large vicinal coupling constant between the olefinic protons (*J*_7,8_ = 15.0 Hz). The NMR interpretation was also confirmed by ESI-MS/MS analysis in which a fragment derived from the cleavage of the C-9 amide bond was observed (See Supporting Information, Figure S1). Thus, the structure of the apliamide D (**4**) was determined as a linear peptide possessing a degraded amino acid.

In addition to the iodinated benzene-containing dipeptides, a dipeptide possessing a different building block was isolated and structurally elucidated. The molecular formula of compound **5** was established as C_21_H_24_N_3_OBr via HRFABMS analysis. A detailed examination of the NMR data revealed that this compound possessed several partial structures that are identical to the other apliamides, as follows: propionamide (C-7–C-9), the phenethylamine moiety (C-1′–C-8′) and the *N*,*N*-dimethyl group (8-NMe_2_) (Table 3). Therefore, the structural difference must occur at the diiodomethoxybenzene moiety, which was replaced with an aromatic moiety having C_8_H_5_NBr as part of its formula.

The new moiety was thought to be a bromoindole because of the characteristic quaternary carbon at δ_C_ 138.9 (C-7a) and a singlet proton at δ_H_ 7.16 (H-2) in the NMR data [26]. This interpretation was fully confirmed by the combined 2D NMR data, including the long-range correlations between the aromatic protons and the neighboring carbons in the HMBC data. The placement of bromine at C-6 of the indole was accomplished using the ABX-type proton coupling pattern of H-4, H-5 and H-7, in addition to the HMBC correlations at H-4 and H-7 with C-6. Similarly the direct linkage between the C-8 ethyl group and the C-3 of the indole was confirmed by the HMBC correlations, as follows: H-2/C-8; H-8/C-2, C-3, C-3a; H-9/C-3. Further confirmation was provided by the ESI-MS/MS data in which a fragment containing a bromoindole moiety was obtained (See Supporting Information, Figure S1). Thus, the structure of apliamide E (**5**) was determined as a dipeptide possessing a tryptophan-derived unit corresponding to the tyrosine-derived units of other apliamides.

Apliamide E (**5**) contained an asymmetric carbon center at the C-9 of the tryptophan-derived unit. Due to the highly unstable nature of the hydrolysates, however, further chemical analyses were unsuccessful under the diverse reaction conditions [27]. The absolute configuration was finally assigned as l by the comparison of the specific rotation (${\left[\text{α}\right]}_{\text{D}}^{25}$ +5.0) sign with a related tryptophan compound (l-*N,N*-dimethyltryptophan methyl ester, ${\left[\text{α}\right]}_{\text{D}}^{25}$ +65.0) [28].

**Table 3:** NMR Data of Compound **5** in MeOH-*d*_4_.

	5	
Position	δ_C_, Type	δ_H_, mult (*J* in Hz)
2	126.5, CH	7.16, s
3	108.1, C	
3a	127.3, C	
4	120.8, CH	7.47, d (8.5)
5	123.4, CH	7.18, d (8.5, 1.5)
6	116.3, C	
7	115.5, CH	7.55, d (1.5)
7a	138.9, C	
8	25.7, CH_2_	3.29, dd (13.5, 5.0)
		3.43, dd (13.5, 10.0)
9	70.5, CH	3.83, dd (10.0, 5.0)
10	167.9, C	
1′	139.8, C	
2′/6′	129.7, CH	6.90, d (7.5)
3′/5′	129.5, CH	7.15, t (7.5)
4′	127.4, CH	7.12, t (7.5)
7′	35.7, CH_2_	2.25, ddd (14.0, 7.0, 7.0)
		2.34, ddd (14.0, 7.0, 7.0)
8′	41.8, CH_2_	3.19, ddd (13.5, 7.5, 7.0)
		3.22, ddd (13.5, 7.5, 7.0)
9-NMe	42.4, CH_3_ (2C)	2.87, s (6H)

In addition to the apliamide dipeptides, a biogenetically related compound was also isolated. The molecular formula of apliamine A (**6**) was deduced as C_11_H_16_NOI_2_ via HRFABMS analysis. The ^1^H and ^13^C NMR data of this compound were very similar to the diiodomethoxybenzene-containing unit of **2** (Table 4), which was confirmed by the combined 2D NMR analyses. Thus, the structure of apliamine A (**6**) was determined as a new amino acid-derived diiodomethoxyphenethylamine.

**Table 4:** NMR Data of Compound **6** in MeOH-*d*_4_.

	6	
Position	δ_C_, Type	δ_H_, mult (*J* in Hz)
1	137.4, C	
2/6	141.6, CH	7.79, s
3/5	91.4, C	
4	159.8, C	
7	29.8, CH_2_	2.95, t (7.5)
8	59.3, CH_2_	3.31, t (7.5)
4-OMe	61.2, CH_3_	3.78, s
8-NMe	44.0, CH_3_ (2C)	2.91, s (6H)

The ascidian-derived iodinated amino acid metabolites exhibited cytotoxic, antimicrobial and glutathione reductase inhibitory activities [14,15]. In our bioactivity tests (Table 5), compounds **1**–**6** exhibited moderate cytotoxicity (IC_50_ 7.8–21.1 µM) for the K562 leukemia cell-line. Although similar trends were found for the A549 lung cancer cell-line (IC_50_ 8.3–22.8 µM), **1** and **4** were inactive (IC_50_ > 100 µM). All compounds exhibited weak cytotoxicity for MRC5 human lung fibroblast cell-line (IC_50_ > 37.0 µM). Compounds **3** and **6** showed no activity for a normal cell-line (MRC5, IC_50_ > 100 µM) but showed moderate cytotoxicity for cancer cell-lines (K562 and A549, IC_50_ 7.8–22.8 µM). In addition, apliamide D (**4**) displayed significant inhibition for the enzyme Na^+^/K^+^-ATPase, (IC_50_ 3.2 µM), which was comparable to ouabain (IC_50_ 6.5 µM). Interestingly, **4** was a strong inhibitor against Na^+^/K^+^-ATPase even it showed poor cytotoxic effect on the normal (MRC5, IC_50_ > 100 µM) and cancer cell lines (K562 and A549, IC_50_ 18.2 and >100 µM, respectively). None of these compounds exhibited significant antibacterial activities against the diverse Gram-positive and Gram-negative strains (MIC > 100 µg/mL) or inhibition against the enzymes sortase A and isocitrate lyase, which are key enzymes in bacterial metabolism (See Supporting Information, Table S1).

**Table 5:** The results of bioactivity tests.

	K562	A549	MRC5	Na^+^/K^+^-ATPase
Compound	IC_50_ (µM)			
**1**	14.3	>100	59.2	163.0
**2**	21.1	10.8	73.7	>200
**3**	10.4	13.4	>100	>200
**4**	18.2	>100	>100	3.2
**5**	7.8	22.8	37.0	>200
**6**	19.7	8.3	>100	189.2
Doxorubicin	1.2	1.4	9.8	
Ouabain				6.5

## 3. Experimental Section

### 3.1. General Experimental Procedures

Optical rotations were measured on a JASCO P-1020 polarimeter using a 1 cm cell. UV spectra were acquired with a Hitachi U-3010 spectrophotometer. IR spectra were recorded on a JASCO 4200 FT-IR spectrometer using a ZnSe cell. NMR spectra were recorded in MeOH-*d*_4_ and CDCl_3_ solutions containing Me_4_Si as an internal standard on Bruker Avance 600, 500 and 400 spectrometers. Proton and carbon NMR spectra were measured at 600 and 150 MHz (**1**, **3**, **4**, and **6**), 500 and 125 MHz (**5**) or 400 and 100 MHz (**2**), respectively (See Supporting Information, Figures S2–S31). The high resolution FAB mass spectrometric data were obtained at the Korea Basic Science Institute (Daegu, Korea) and acquired using a JEOL JMS 700 mass spectrometer with *meta*-nitrobenzyl alcohol (NBA) as a matrix for the FABMS. The low-resolution ESIMS data were recorded on an Agilent Technologies 6130 Quadrupole mass spectrometer with an Agilent Technologies 1200 series HPLC. The ESI-QTOF-MS/MS was performed on an Agilent Technologies 6530 Accurate-Mass Q-TOF LC/MS spectrometer with an Agilent Technologies 1260 series HPLC. The semi-preparative HPLC was performed on a Spectrasystem p2000 equipped with a refractive index detector (Spectrasystem RI-150) and a YMC ODS-A column (10 × 250 mm). All of the solvents used were spectroscopic grade or were glass-distilled prior to use.

### 3.2. Animal Materials

Specimens of *Aplidium* sp. (sample number 12CH-18) were manually collected with scuba equipment at a depth of 20 m off the coast of Chuja-do, Korea, on 10 October 2012. The colony was gelatinous and spherical in shape, 35 mm thick, 102 mm in maximum dimension, and attached via a large portion of the basal surface. The colony was reddish-orange live and yellowish-beige in ethanol. The zooids were beige in color and were situated perpendicularly during the test. The zooids were 1.25–9.97 mm in length, of which the thorax, abdomen and posterior abdomen were 0.24–0.58, 0.23–1.39 and 0.78–8.00 mm, respectively. The thorax was short with 13–14 stigma rows and an atrial tongue cleft. The gut loop was vertical and U-formed. The stomach was barrel-shaped, orange in color, and had 18–20 longitudinal folds. The posterior abdomen was very long and thread-like and occupied more than half of the body length. However, the lack of gonads and larvae prevented adequate species-level identification. The voucher specimens were deposited at the Natural History Museum, Ewha Womans University, under the curatorship of B.J.R.

### 3.3. Extraction and Isolation

Freshly collected specimens were immediately frozen and stored at −25 °C until use. The lyophilized specimens were macerated and repeatedly extracted with MeOH (3 L × 3) and CH_2_Cl_2_ (3 L × 2). The combined extracts (38.70 g) were successively partitioned between H_2_O (25.62 g) and *n*-BuOH (11.50 g); the latter fraction was repartitioned between H_2_O and MeOH (15:85) (7.09 g) and *n*-hexane (3.99 g). The former layer was separated by C_18_ reversed-phase flash chromatography using sequential mixtures of MeOH and H_2_O (six fractions in gradient, from 50:50 to 0:100), acetone, and, finally, EtOAc as the eluents.

Based on the ^1^H NMR results and the cytotoxicity analyses, the fractions eluted with H_2_O-MeOH (50:50; 1.12 g) and H_2_O-MeOH (20:80; 1.00 g) were selected for separation. The former fraction was separated by reversed-phase semi-preparative HPLC (H_2_O-MeOH, 55:45 with 0.01% TFA) to yield **6**. The latter H_2_O-MeOH (20:80) fraction was separated by reversed-phase HPLC (H_2_O-MeOH, 30:70) to produce, in order of elution, compounds **5**, **1**, **2**, **4**, and **3** as the amorphous solids. All of the isolated compounds showed high purities in the NMR data and were not further purified. The isolated amounts were 32.2, 98.6, 6.3, 3.2, 12.8, and 5.5 mg for **1**–**6**, respectively.

Apliamide A (**1**): Yellow amorphous solid; ${\left[\text{α}\right]}_{\text{D}}^{25}$ +12.3 (*c* 0.50, MeOH); UV (MeOH) λ_max_ (log ε) 205 (2.75), 210 (2.74), 226 (2.71), 244 (2.41), 277 (1.85) nm; IR (ZnSe) ν_max_ 3297, 2932, 1655, 1529, 1460 cm^−1^; ^1^H and ^13^C NMR data, see Table 1; HRFABMS *m*/*z* 564.9851 [M + H]^+^ (calcd for C_19_H_23_N_2_O_2_I_2_, 564.9849).

Apliamide B (**2**): Yellow amorphous solid; ${\left[\text{α}\right]}_{\text{D}}^{25}$ +11.2 (*c* 0.50, MeOH); UV (MeOH) λ_max_ (log ε) 205 (2.77), 226 (2.72), 244 (2.51), 276 (2.01) nm; IR (ZnSe) ν_max_ 3308, 2934, 1658, 1531, 1460 cm^−1^; ^1^H and ^13^C NMR data, see Table 1; HRFABMS *m*/*z* 579.0003 [M + H]^+^ (calcd for C_20_H_25_N_2_O_2_I_2_, 579.0006).

Apliamide C (**3**): Yellow amorphous solid; ${\left[\text{α}\right]}_{\text{D}}^{25}$ +10.3 (*c* 0.50, MeOH); UV (MeOH) λ_max_ (log ε) 205 (2.79), 227 (2.69), 245 (2.49), 282 (2.03) nm; IR (ZnSe) ν_max_ 3297, 2928, 1657, 1530, 1459 cm^−1^; ^1^H and ^13^C NMR data, see Table 2; HRFABMS *m*/*z* 734.9078 [M + H]^+^ (calcd for C_21_H_26_N_2_O_3_I_3_, 734.9084).

Apliamide D (**4**): Yellow amorphous solid; UV (MeOH) λ_max_ (log ε) 205 (2.72), 245 (2.74), 287 (2.49) nm; IR (ZnSe) ν_max_ 3285, 2932, 1747, 1658, 1565, 1461 cm^−1^; ^1^H and ^13^C NMR data, see Table 2; HRFABMS *m*/*z* 533.9424 [M + H]^+^ (calcd for C_18_H_18_NO_2_I_2_, 533.9427).

Apliamide E (**5**): Yellow amorphous solid; ${\left[\text{α}\right]}_{\text{D}}^{25}$ +5.0 (*c* 0.50, MeOH); UV (MeOH) λ_max_ (log ε) 205 (2.62), 228 (1.63), 280 (2.23) nm; IR (ZnSe) ν_max_ 3284, 2929, 1655, 1524, 1454 cm^−1^; ^1^H and ^13^C NMR data, see Table 3; HRFABMS *m*/*z* 414.1190 [M + H]^+^ (calcd for C_21_H_25_N_3_O^79^Br, 414.1181).

Apliamine A (**6**): White amorphous solid; UV (MeOH) λ_max_ (log ε) 205 (2.82), 227 (2.87), 243 (2.67), 282 (2.30) nm; IR (ZnSe) ν_max_ 3262, 2933, 1622, 1460 cm^−1^; ^1^H and ^13^C NMR data, see Table 4; HRFABMS *m*/*z* 431.9327 [M + H]^+^ (calcd for C_11_H_16_NOI_2_, 431.9321).

### 3.4. Advanced Marfey’s Analysis of Compound **1**

Apliamide A (**1**, 1.2 mg) was dissolved in 12 N HCl (0.5 mL) and heated at 110 °C for 16 h. The solution and traces of HCl were removed by repeated drying under vacuum with distilled water. To the divided hydrolysate (0.6 mg each), 1 N NaHCO_3_ (100 µL) and 1% l- or d-FDAA (50 µL) in acetone were added. The mixture was stirred at 80 °C for 12 min. After quenching the reaction by the addition of 2 N HCl (50 µL), the residue was analyzed using ESI-LC/MS with a Phenomenex Luna C18 (5 µm, 4.6 mm × 150 mm) analytical column. The mobile phase flow rate was 0.7 mL/min and a gradient elution of A (water with 0.1% formic acid) and B (MeCN with 0.1% formic acid) was used (0 min, 10% B; 40 min, 70% B in A *v*/*v*). The retention times of the l- and d-FDAA-derivatized hydrolysates were 30.03 and 31.04 min, respectively, leading to assignment of the l configuration.

### 3.5. Biological Assays

The cytotoxicity assays were performed in accordance with the literature protocols [29]. The Na^+^/K^+^-ATPase, isocitrate lyase, and sortase A inhibition assays were performed according to previously described methods [30,31,32]. The antimicrobial assays were performed according to the method described previously [33].

## 4. Conclusions

Six new metabolites, four iodobenzene containing dipeptides (**1**–**4**), a related bromotryptophan containing dipeptide (**5**), and an iodobenzene amine (**6**), were isolated from the ascidian *Aplidium* sp. collected from Korean waters. These compounds possessed structural novelty at their iodinated and brominated amino acid units. Several of these compounds exhibited moderate cytotoxicity for the K562 and A549 cell-lines. Additionally, apliamide D (**4**) exhibited significant inhibition for the enzyme Na^+^/K^+^-ATPase.

## Supplementary Files

Supplementary File 1
