# Supplementary material for: Amino Acid-Derived Metabolites from the Ascidian Aplidium sp
Source: Mar Drugs. 2015 Jun 16;13(6):3836–48. doi: 10.3390/md13063836 (PMC4483659; doi:10.3390/md13063836)
Supplement: Supplementary File 1 [file marinedrugs-13-03836-s001.pdf]

Supplementary Information

ESI-Q-TOF-MS/MS fragmentations of compounds **1**-**5** Figure S1
The ^{1}H NMR (600 MHz, MeOH-d_4) spectrum of apliamide **A** (**1**) Figure S2
The ^{13}C NMR (150 MHz, MeOH-d_4) spectrum of apliamide **A** (**1**) Figure S3
The COSY (600 MHz, MeOH-d_4) spectrum of apliamide A (**1**) Figure S4
The *g*HSQC (600 MHz, MeOH-d_4) spectrum of apliamide A (**1**) Figure S5
The *g*HMBC (600 MHz, MeOH-d_4) spectrum of apliamide A (**1**) Figure S6
The ^{1}H NMR (400 MHz, MeOH-d_4) spectrum of apliamide B (**2**) Figure S7
The ^{13}C NMR (100 MHz, MeOH-d_4) spectrum of apliamide B (**2**) Figure S8
The COSY (400 MHz, MeOH-d_4) spectrum of apliamide B (**2**) Figure S9
The *g*HSQC (400 MHz, MeOH-d_4) spectrum of apliamide B (**2**) Figure S10
The *g*HMBC (400 MHz, MeOH-d_4) spectrum of apliamide B (**2**) Figure S11
The ^{1}H NMR (600 MHz, MeOH-d_4) spectrum of apliamide C (**3**) Figure S12
The ^{13}C NMR (150 MHz, MeOH-d_4) spectrum of apliamide C (**3**) Figure S13
The COSY (600 MHz, MeOH-d_4) spectrum of apliamide C (**3**) Figure S14
The *g*HSQC (600 MHz, MeOH-d_4) spectrum of apliamide C (**3**) Figure S15
The *g*HMBC (600 MHz, MeOH-d_4) spectrum of apliamide C (**3**) Figure S16
The ^{1}H NMR (600 MHz, $CDCl_3$) spectrum of apliamide D (**4**) Figure S17
The ^{13}C NMR (150 MHz, $CDCl_3$) spectrum of apliamide D (**4**) Figure S18
The COSY (600 MHz, $CDCl_3$) spectrum of apliamide D (**4**) Figure S19
The *g*HSQC (600 MHz, $CDCl_3$) spectrum of apliamide D (**4**) Figure S20
The *g*HMBC (600 MHz, $CDCl_3$) spectrum of apliamide D (**4**) Figure S21
The ^{1}H NMR (500 MHz, MeOH-d_4) spectrum of apliamide E (**5**) Figure S22
The ^{13}C NMR (125 MHz, MeOH-d_4) spectrum of apliamide E (**5**) Figure S23
The COSY (500 MHz, MeOH-d_4) spectrum of apliamide E (**5**) Figure S24
The *g*HSQC (500 MHz, MeOH-d_4) spectrum of apliamide E (**5**) Figure S25
The *g*HMBC (500 MHz, MeOH-d_4) spectrum of apliamide E (**5**) Figure S26
The ^{1}H NMR (600 MHz, MeOH-d_4) spectrum of apliamine A (**6**) Figure S27
The ^{13}C NMR (150 MHz, MeOH-d_4) spectrum of apliamine A (**6**) Figure S28
The COSY (600 MHz, MeOH-d_4) spectrum of apliamine A (**6**) Figure S29
The *g*HSQC (600 MHz, MeOH-d_4) spectrum of apliamine A (**6**) Figure S30
The *g*HMBC (600 MHz, MeOH-d_4) spectrum of apliamine A (**6**) Figure S31
The result of antibacterial and enzyme inhibition test. Table S1

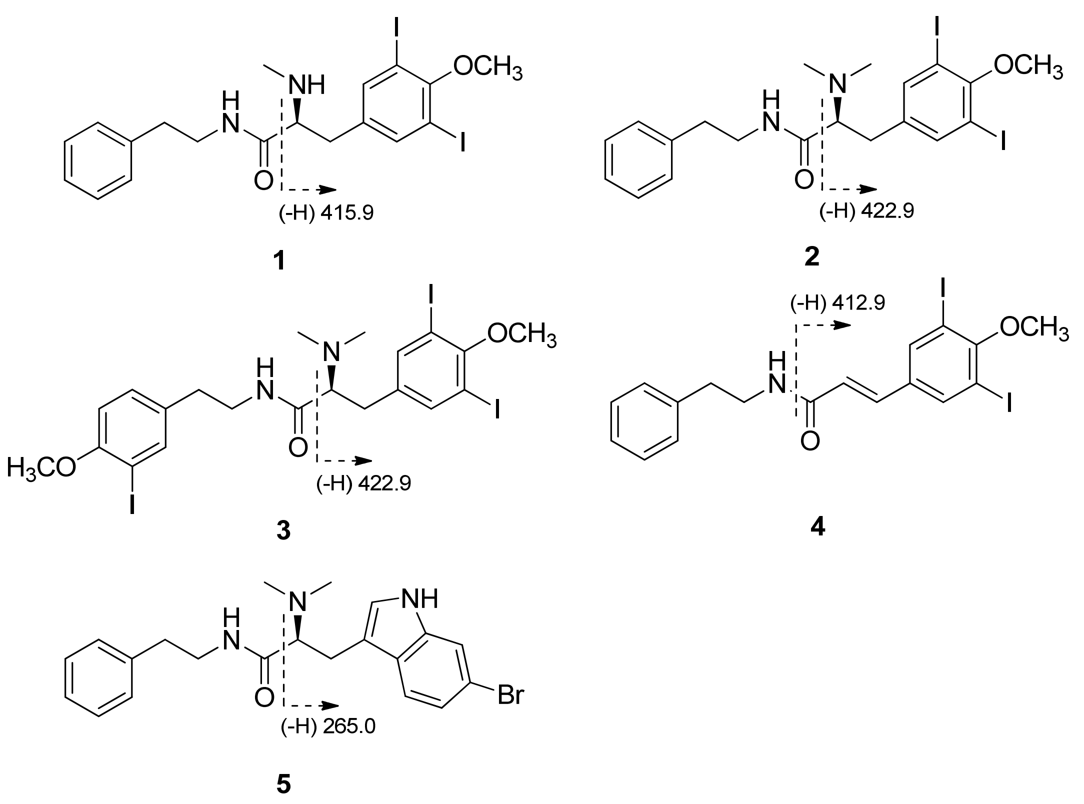

Figure S1. ESI-Q-TOF-MS/MS fragmentations of compounds **1**–**5**.

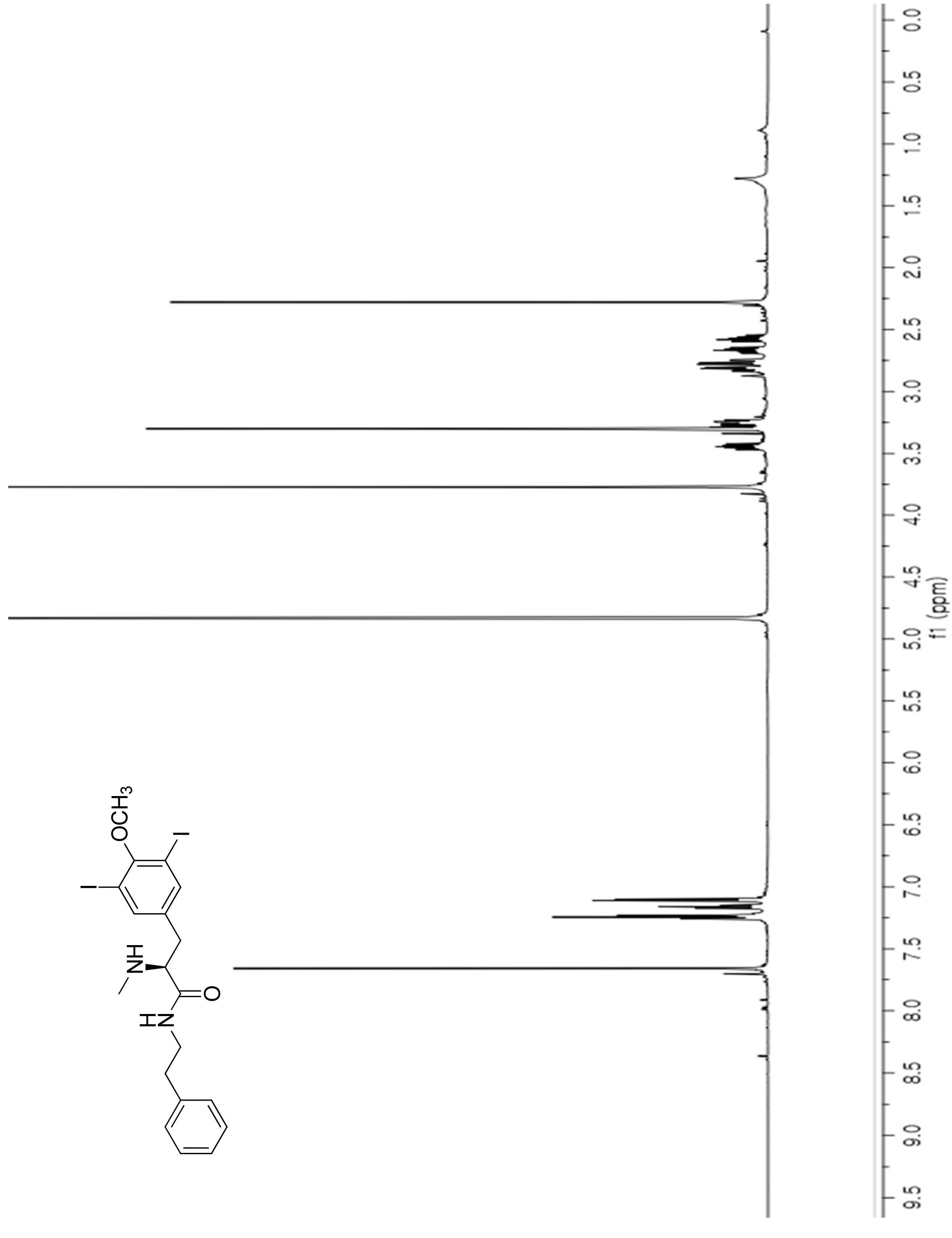

Figure S2. The ^{1}H NMR (600 MHz, MeOH-*d*4) spectrum of apliamide A (**1**).

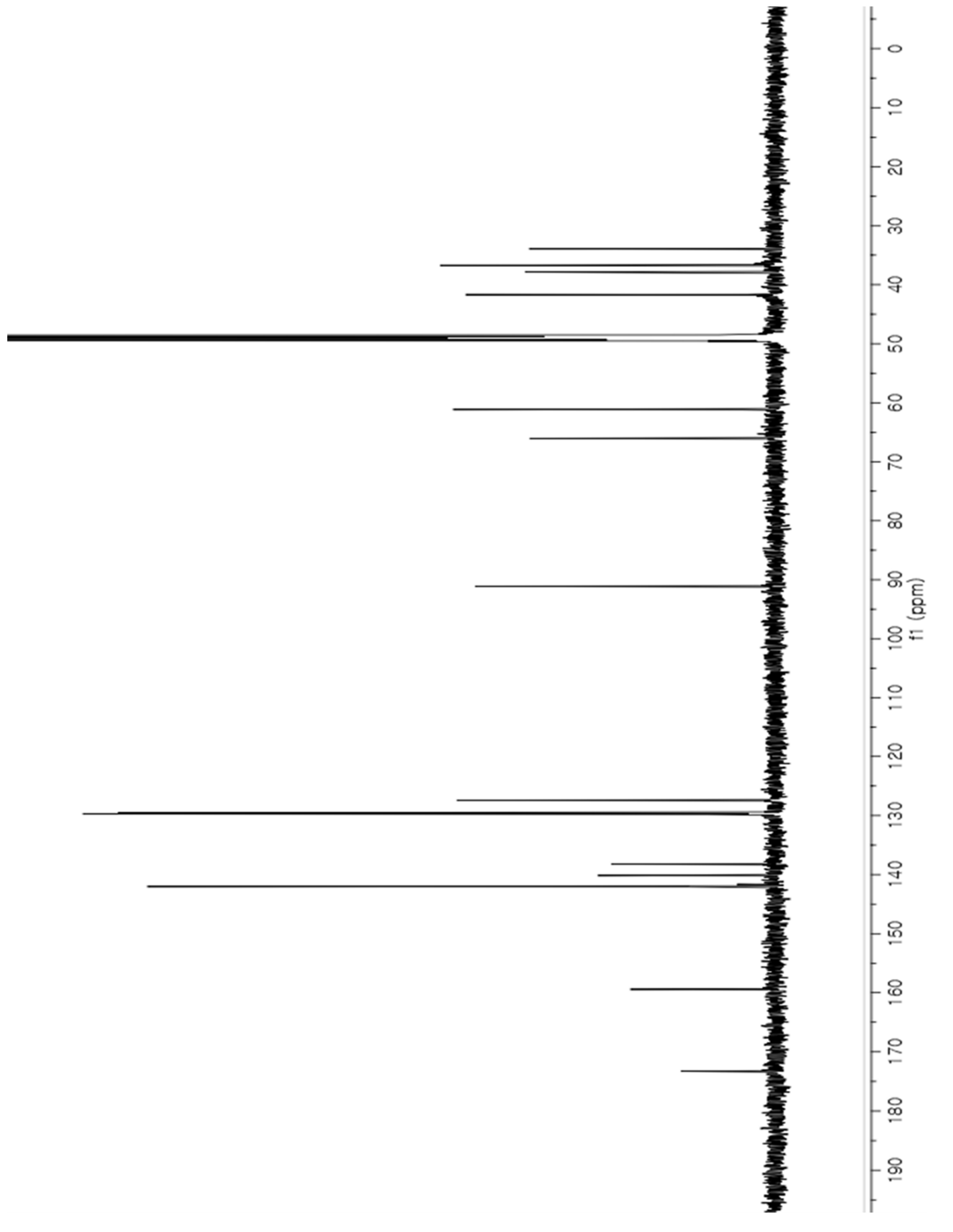

Figure S3. The ^{13}C NMR (150 MHz, MeOH-d_4) spectrum of apliamide A (**1**).

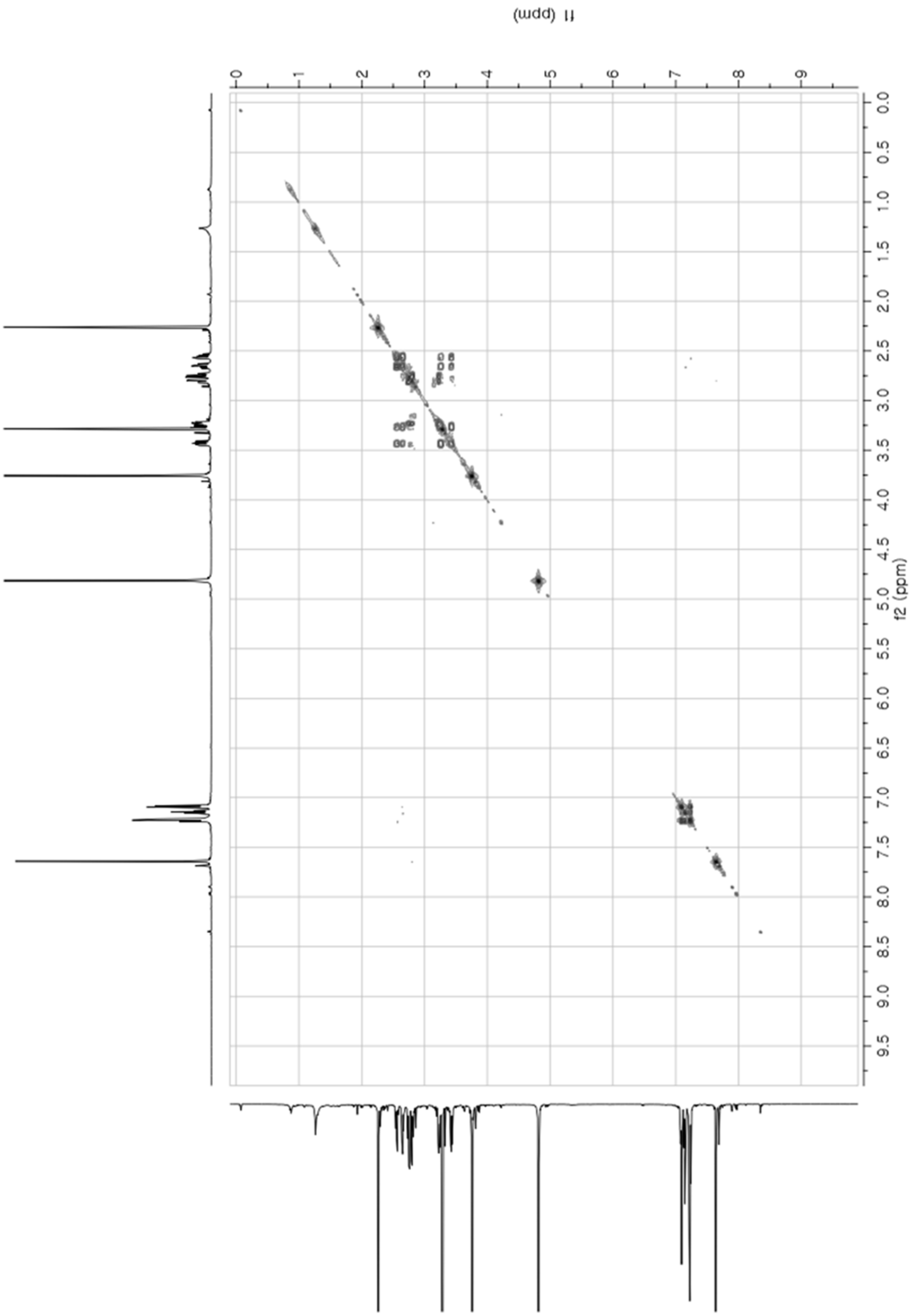

Figure S4. The COSY (600 MHz, MeOH-d_4) spectrum of apliamide A (**1**)

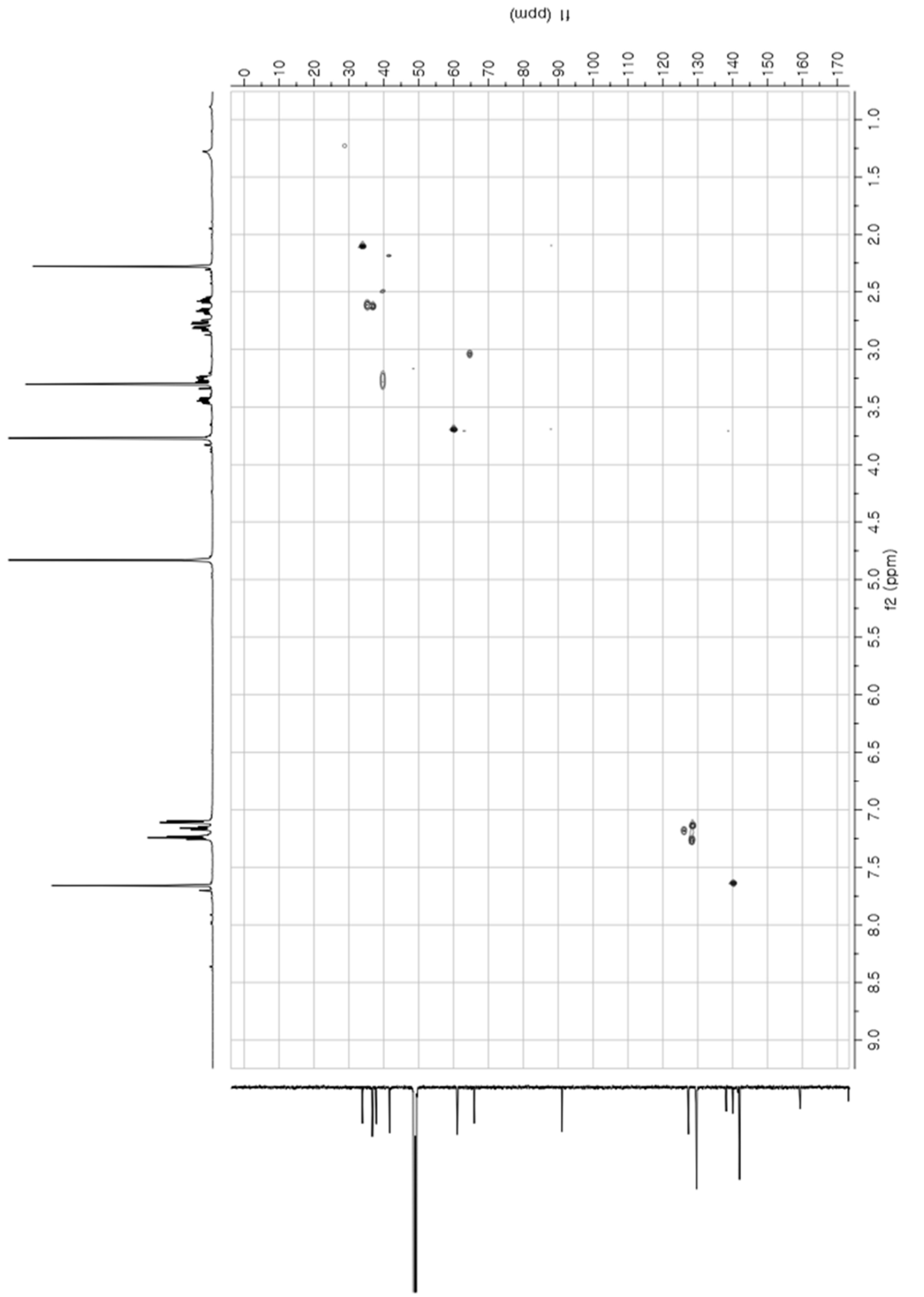

Figure S5. The *g*HSQC (600 MHz, MeOH-d_4) spectrum of apliamide A (**1**).

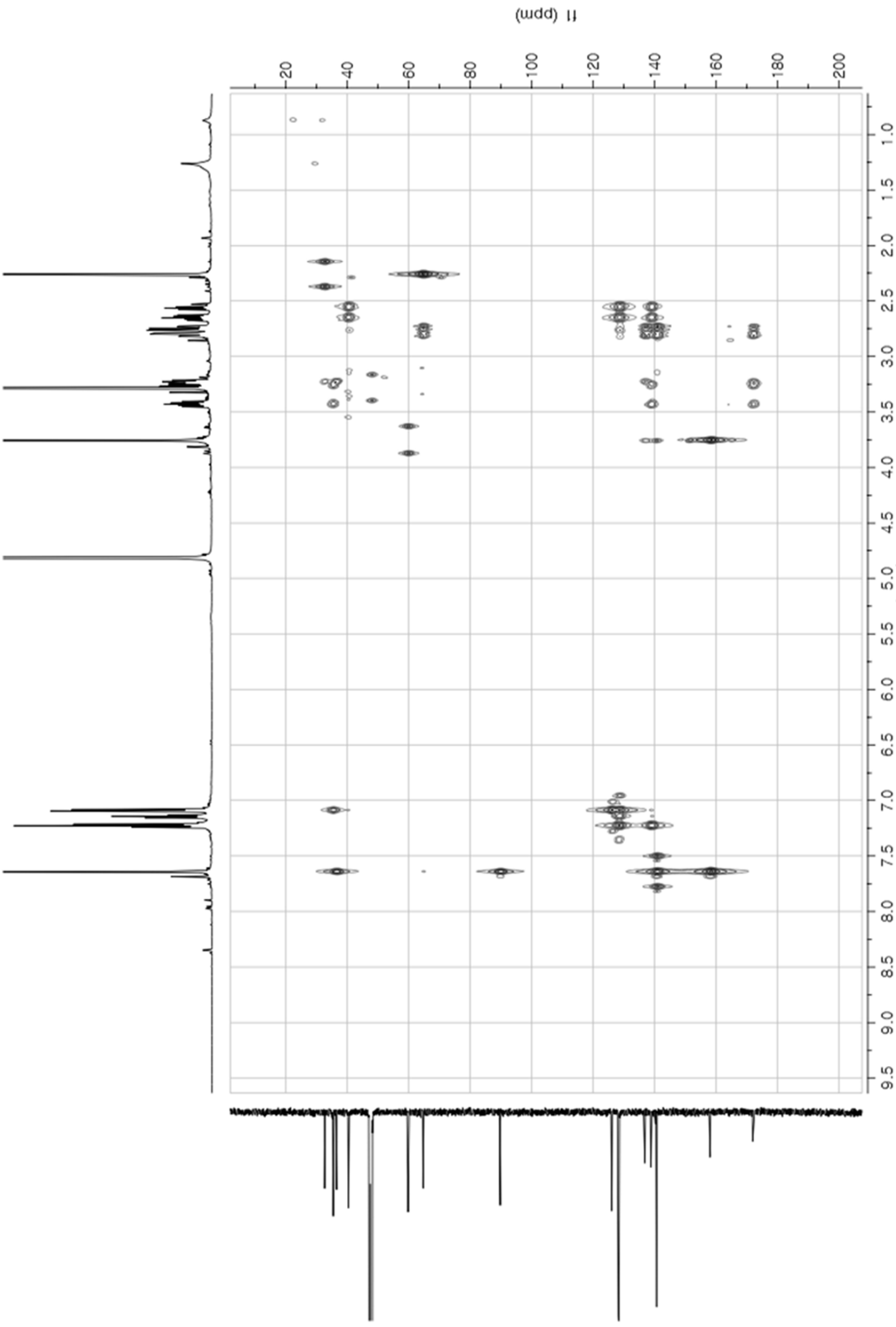

Figure S6. The *g*HMBC (600 MHz, MeOH-d_4) spectrum of apliamide A (**1**).

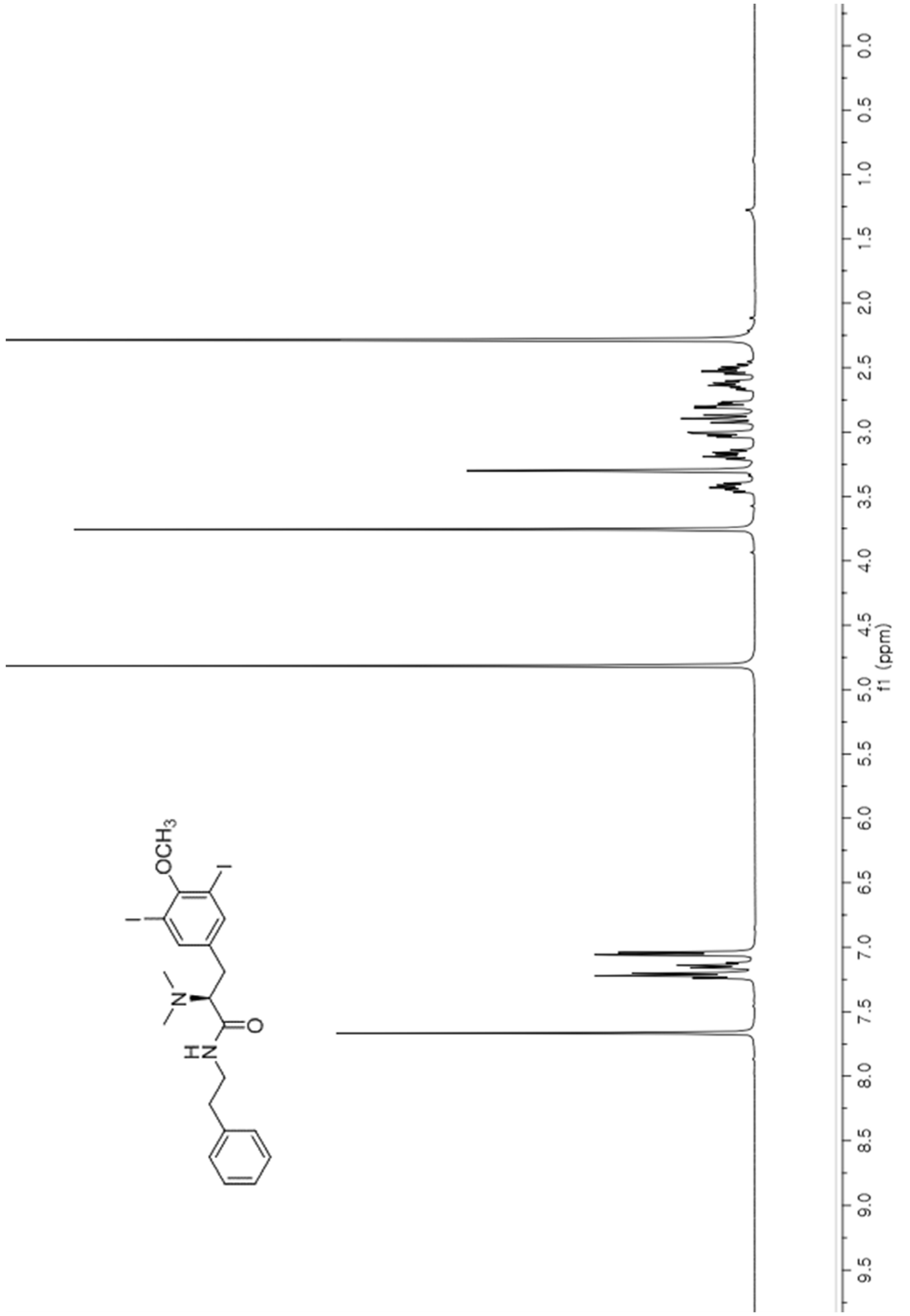

Figure S7. The ^{1}H NMR (400 MHz, MeOH-d_4) spectrum of apliamide B (**2**).

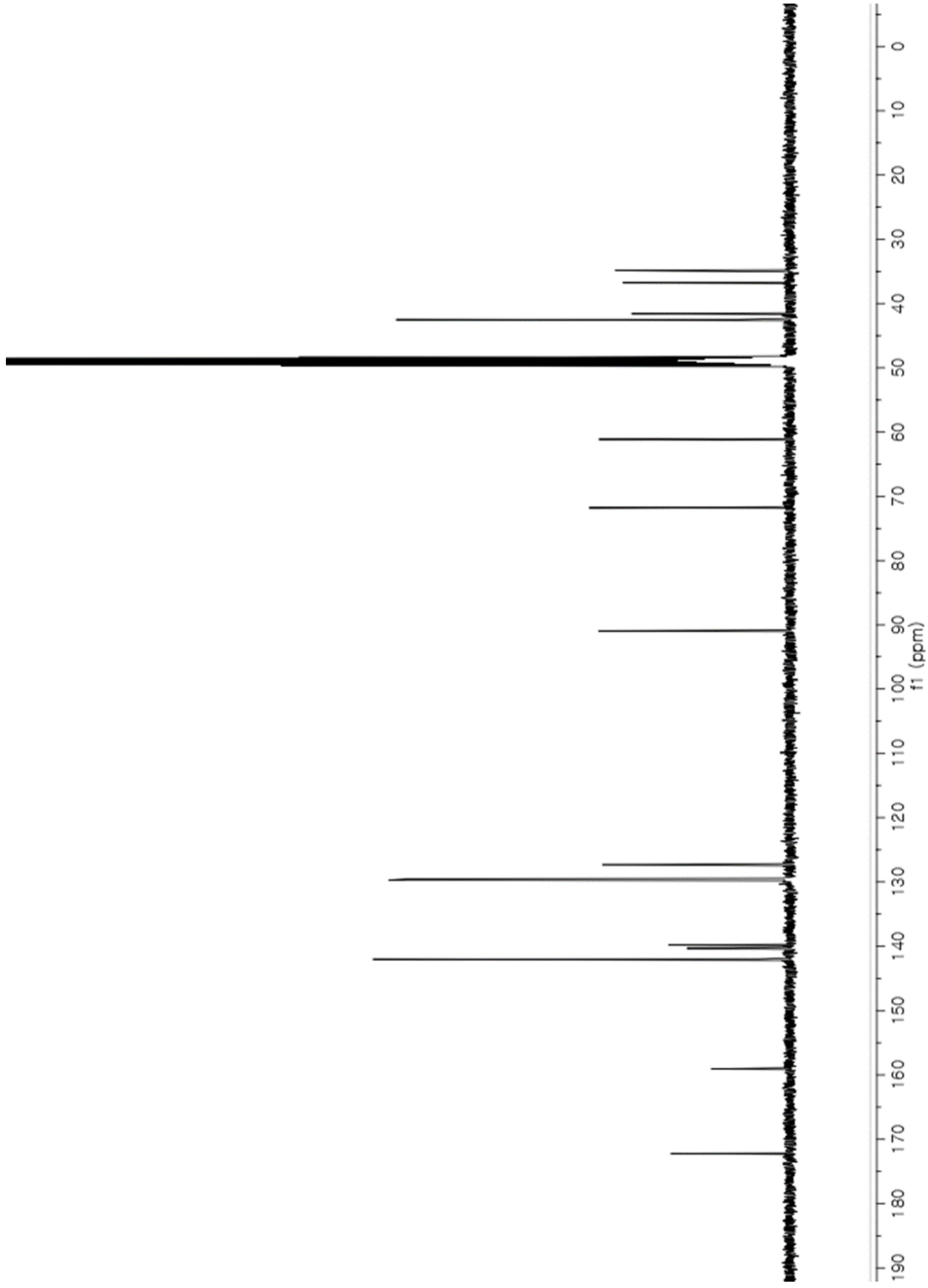

Figure S8. The ^{13}C NMR (100 MHz, MeOH-d_4) spectrum of apliamide B (**2**).

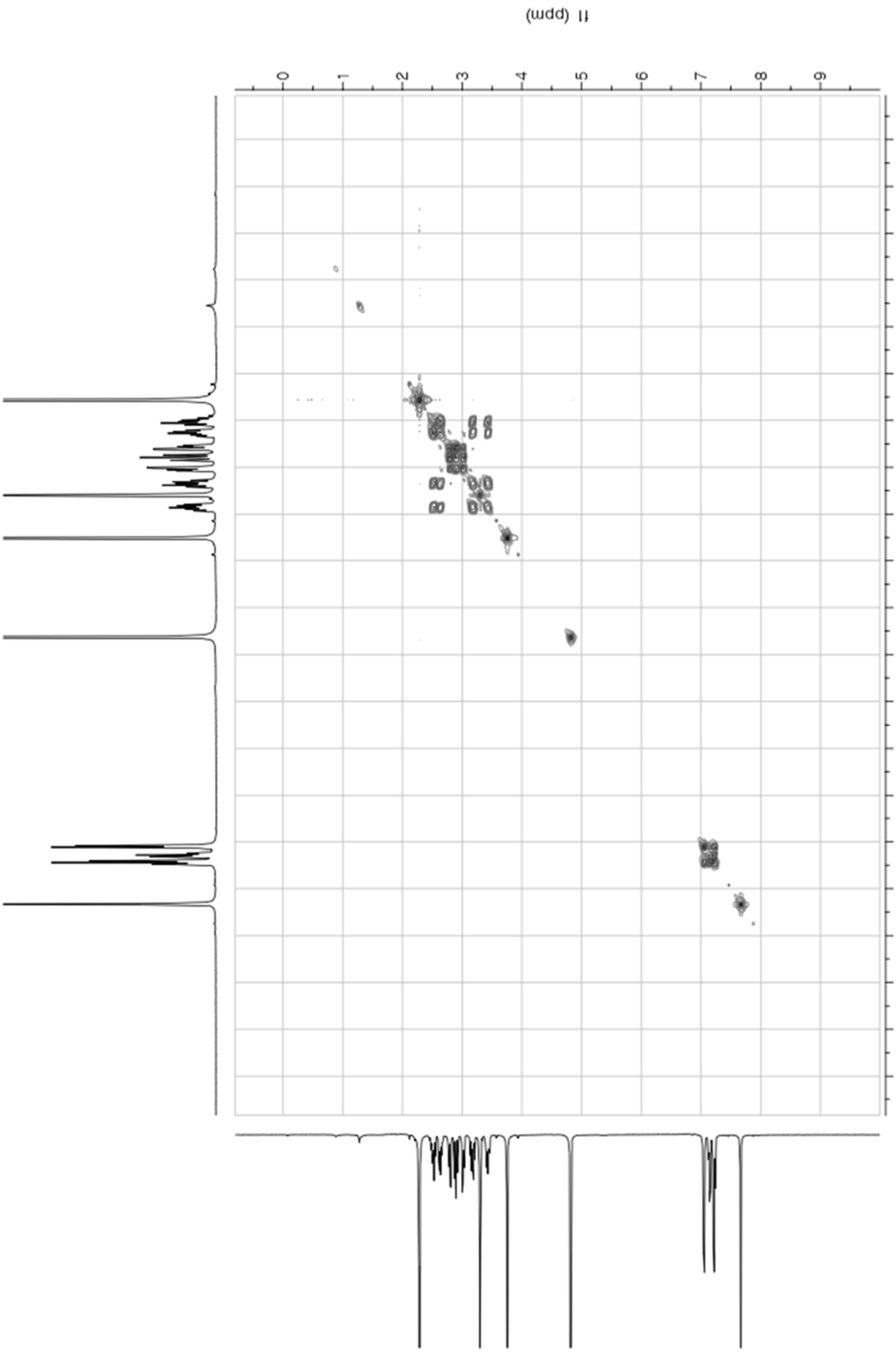

Figure S9. The COSY (400 MHz, MeOH-d_4) spectrum of apliamide B (**2**).

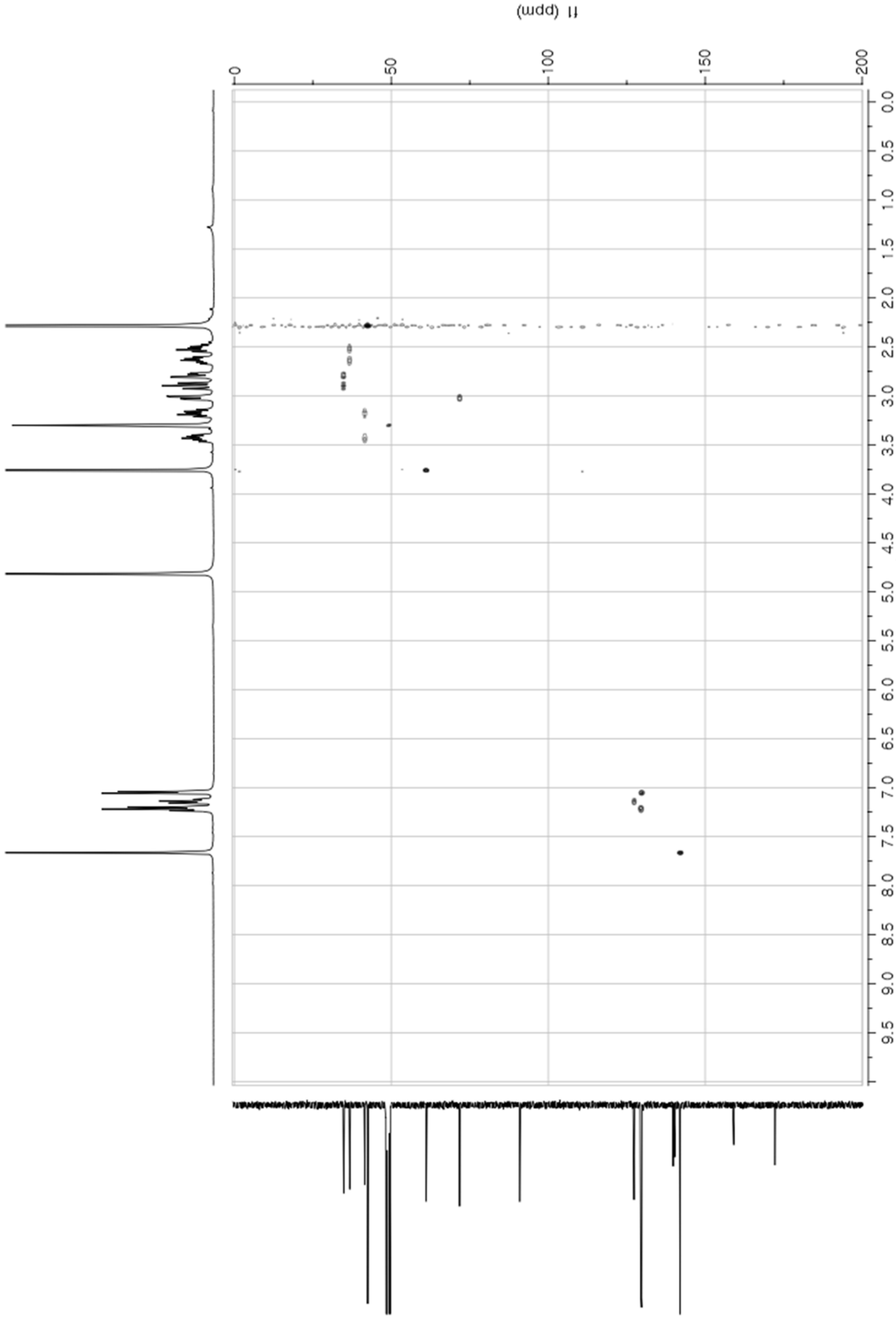

Figure S10. The *g*HSQC (400 MHz, MeOH-d_4) spectrum of apliamide B (**2**).

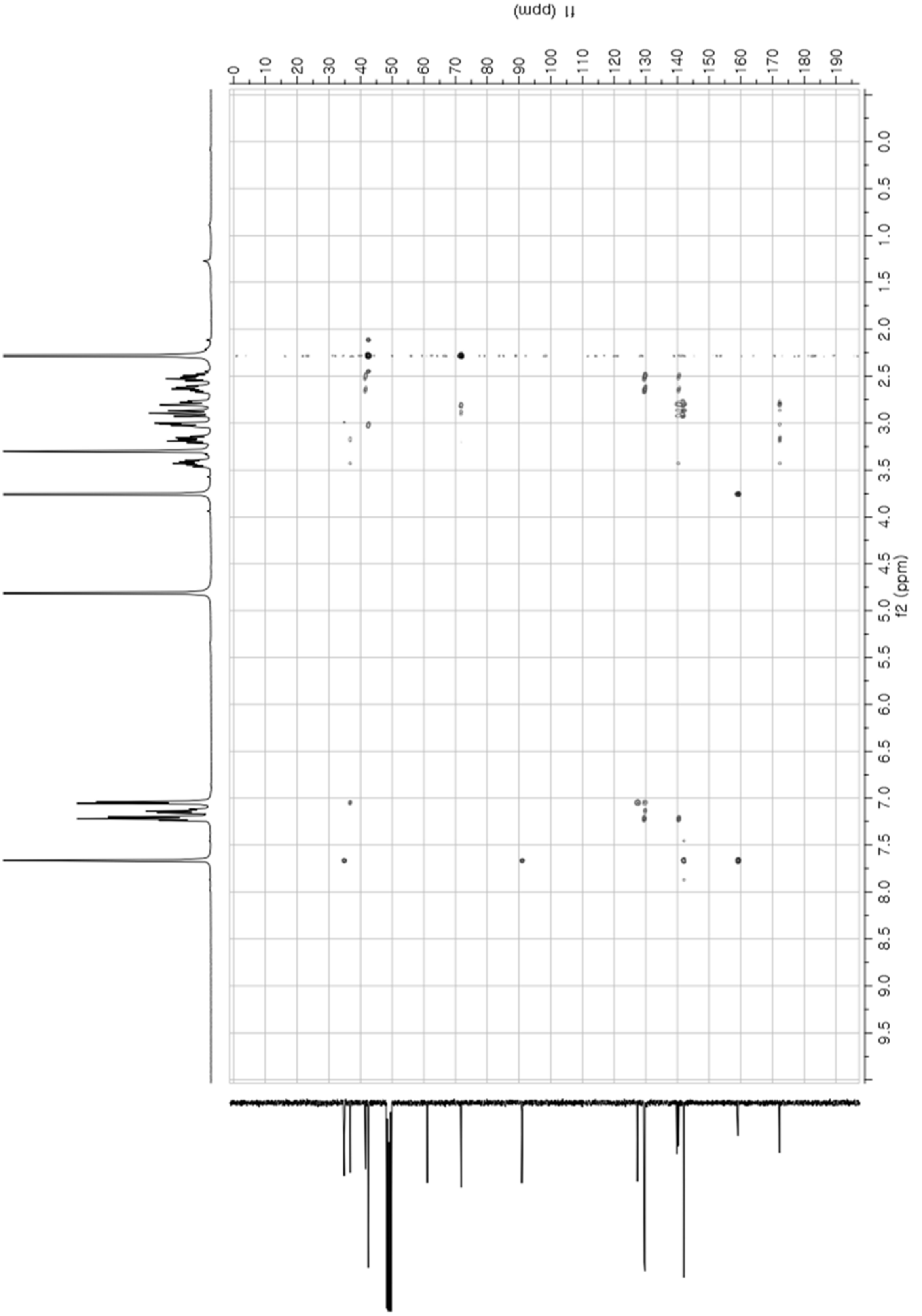

Figure S11. The *g*HMBC (400 MHz, MeOH-d_4) spectrum of apliamide B (**2**).

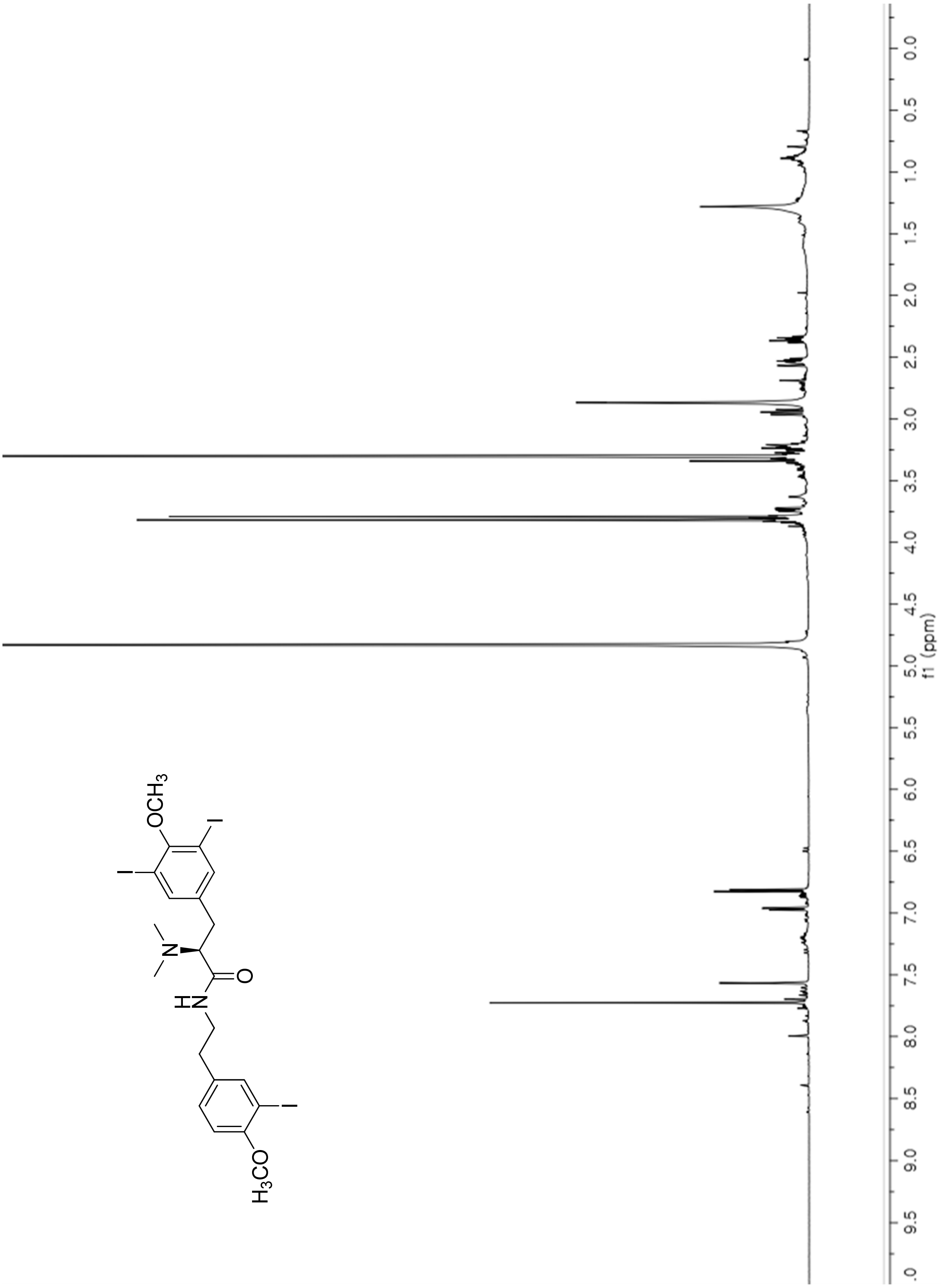

Figure S12. The ^{1}H NMR (600 MHz, MeOH-d_4) spectrum of apliamide C (**3**).

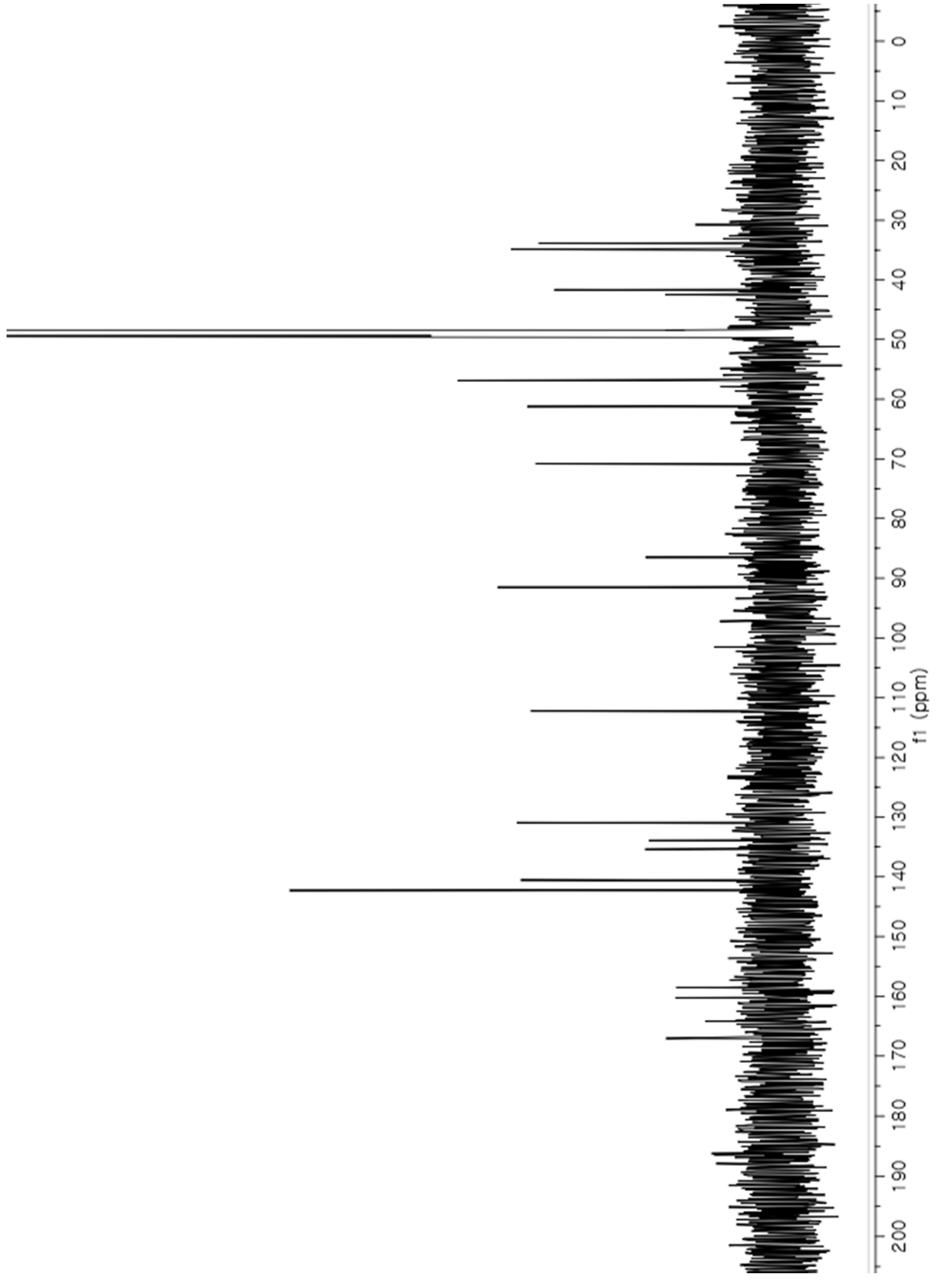

Figure S13. The ^{13}C NMR (150 MHz, MeOH-d_4) spectrum of apliamide C (**3**).

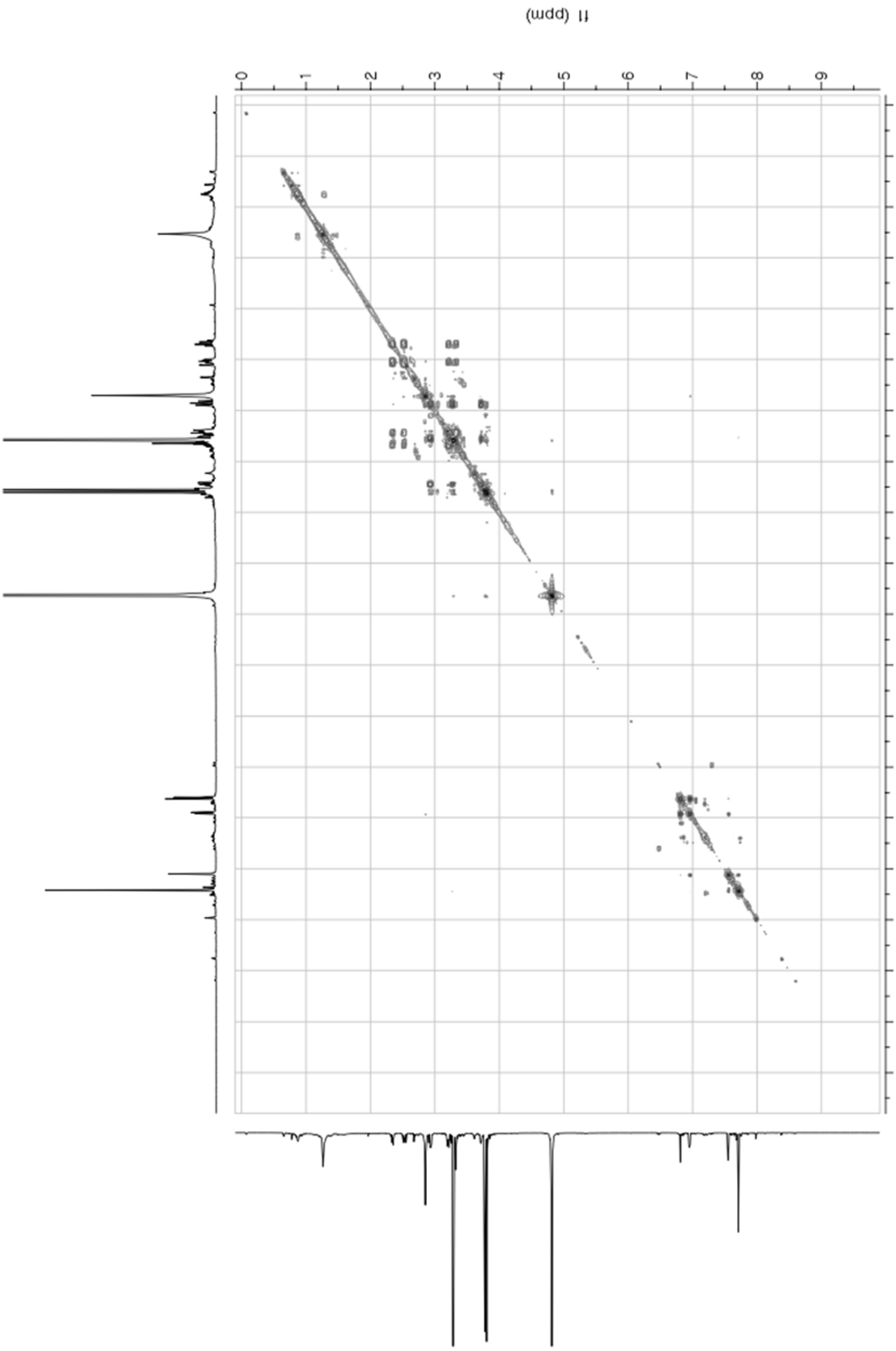

Figure S14. The COSY (600 MHz, MeOH-d_4) spectrum of apliamide C (**3**).

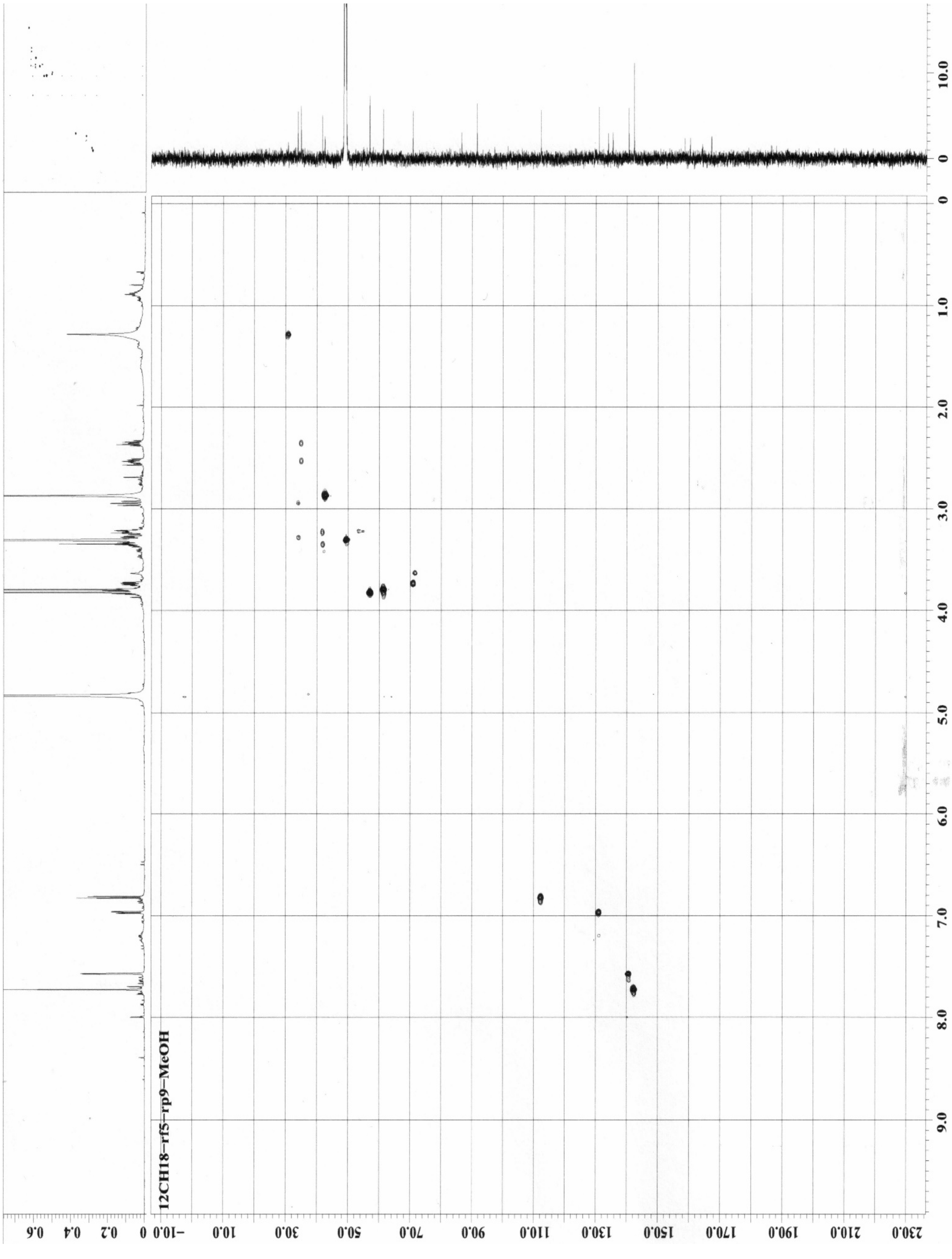

Figure S15. The *g*HSQC (600 MHz, MeOH-*d*4) spectrum of apliamide C (**3**).

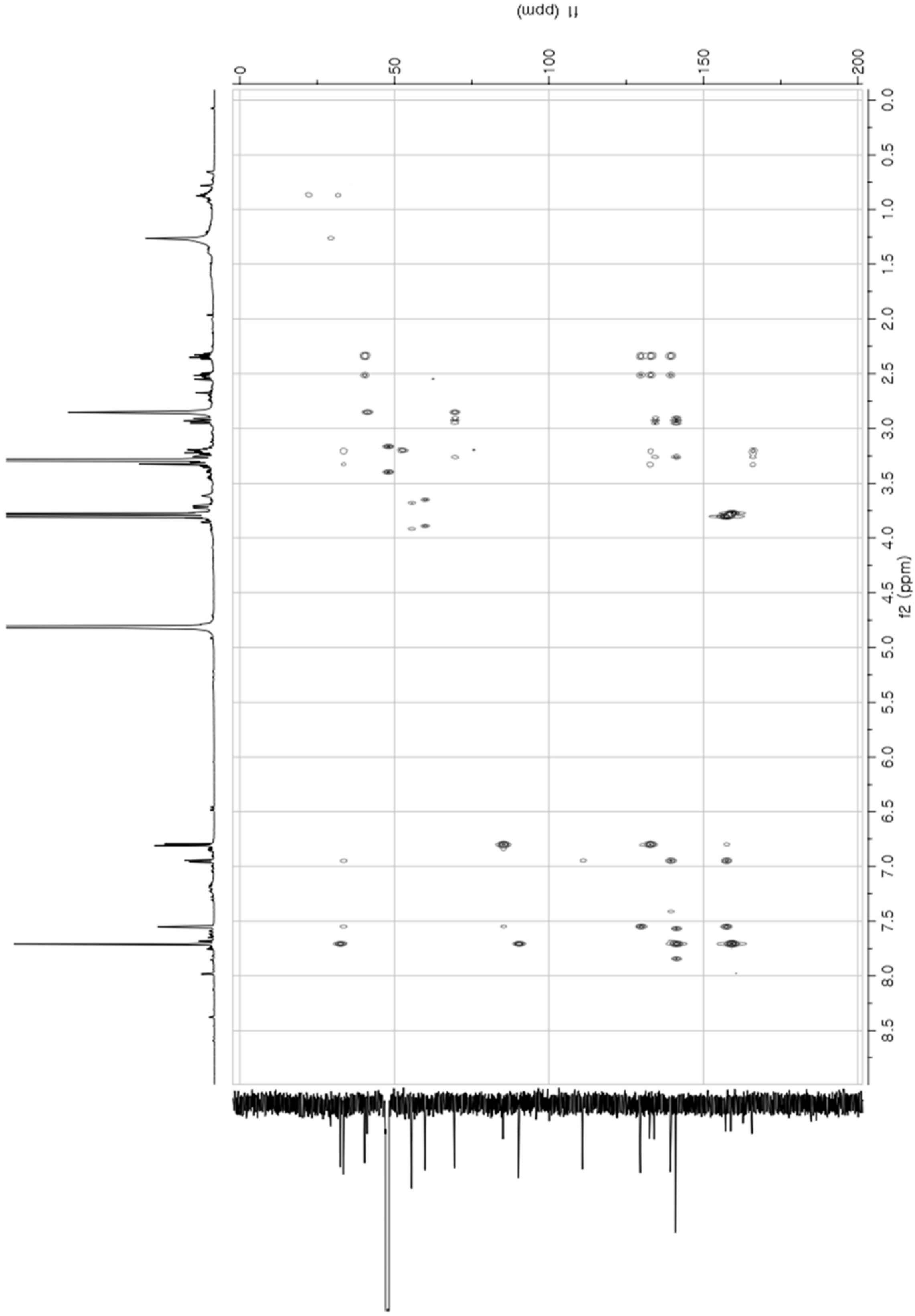

Figure S16. The *g*HMBC (600 MHz, MeOH-d_4) spectrum of apliamide C (**3**).

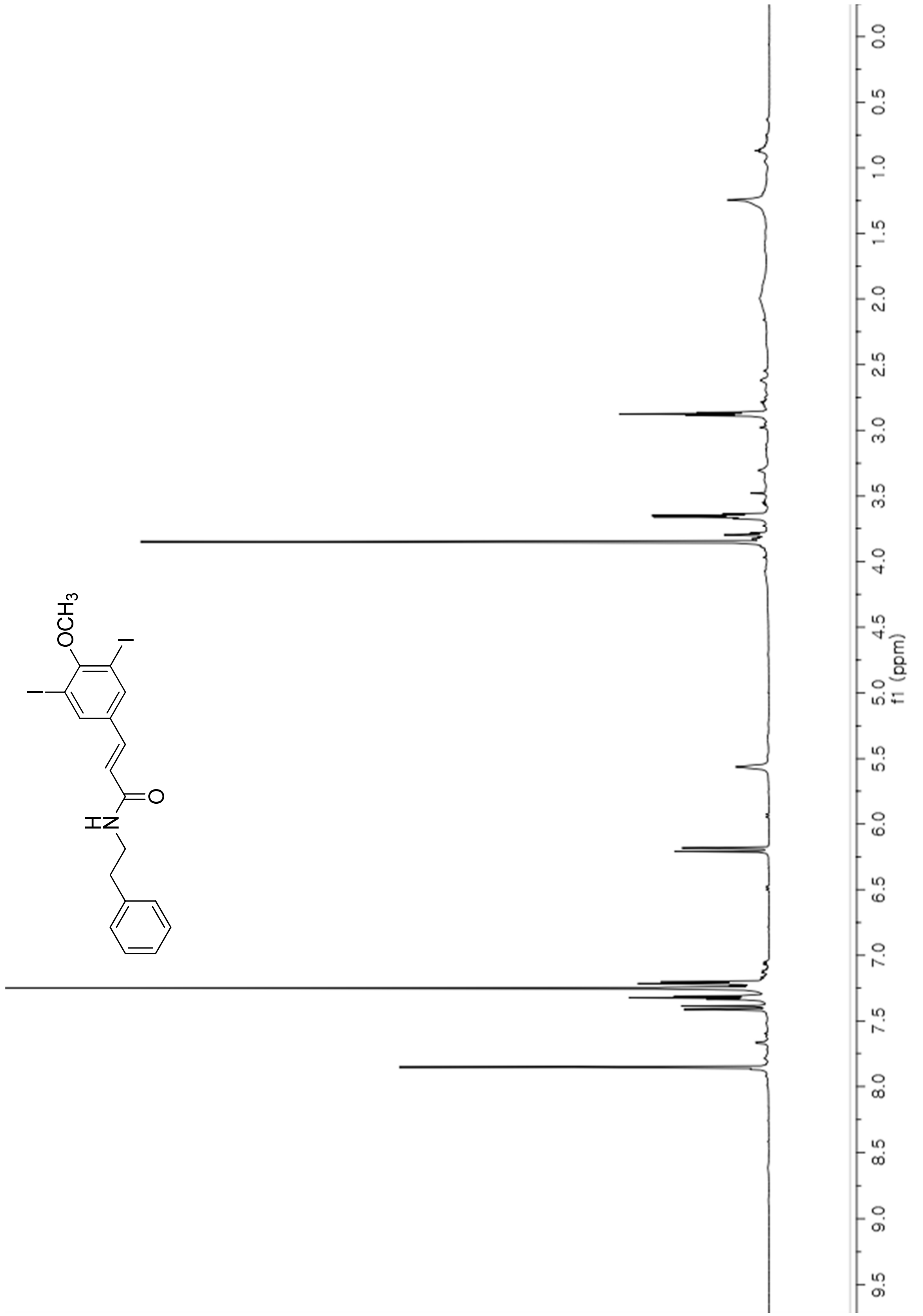

Figure S17. The ^{1}H NMR (600 MHz, $CDCl_3$) spectrum of apliamide D (**4**).

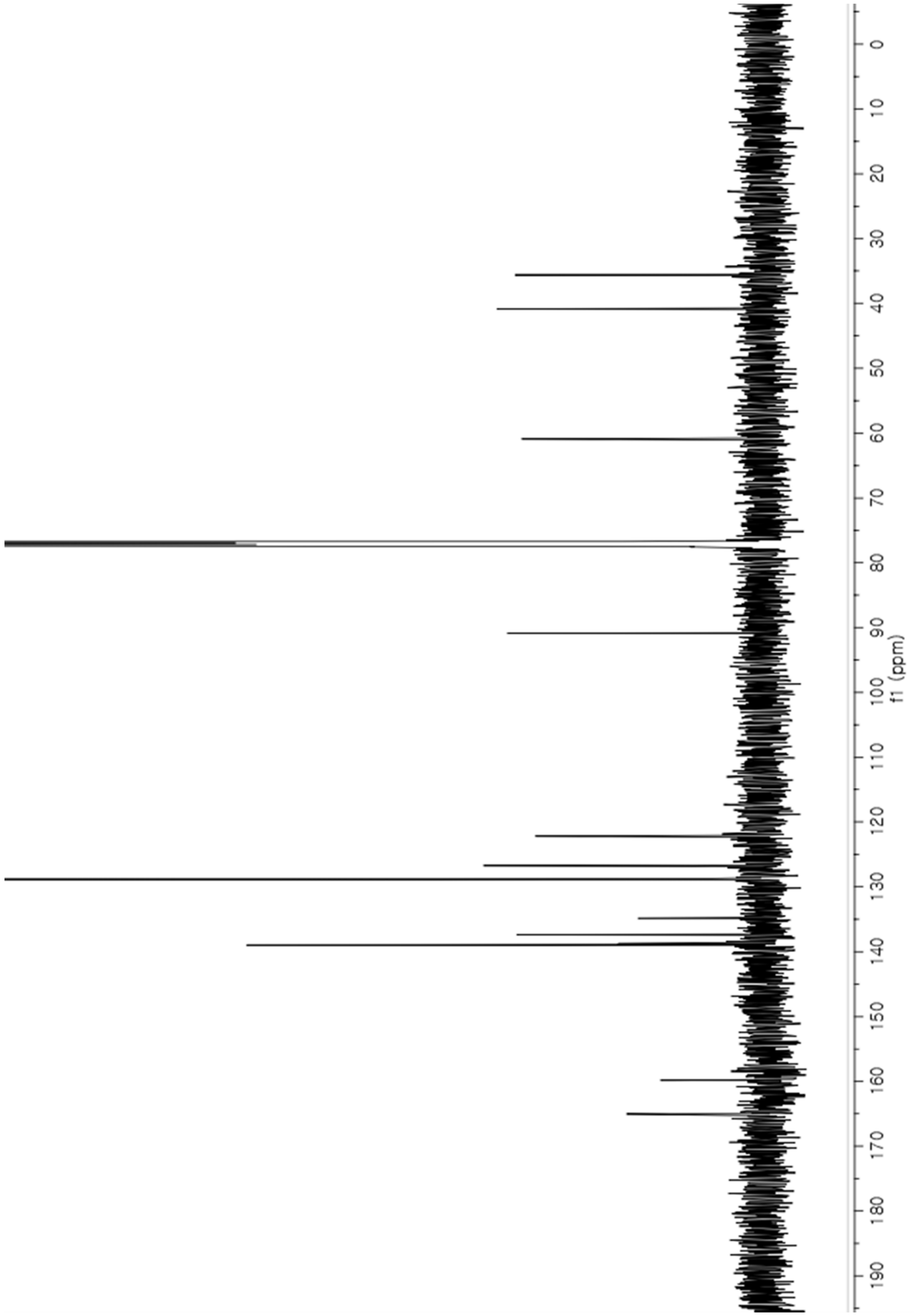

Figure S18. The ^{13}C NMR (150 MHz, $CDCl_3$) spectrum of apliamide D (**4**).

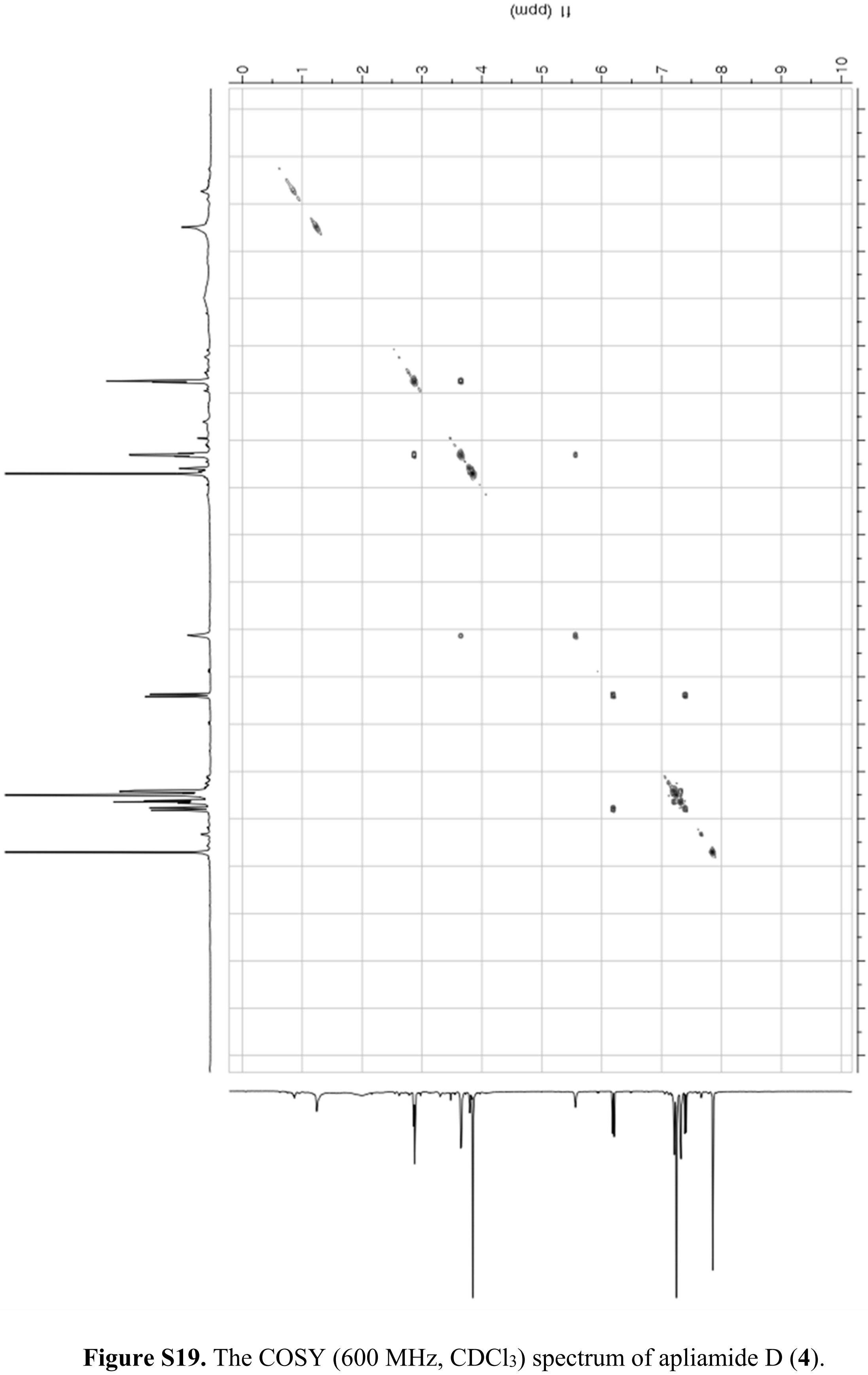

Figure S19. The COSY (600 MHz, $CDCl_3$) spectrum of apliamide D (**4**).

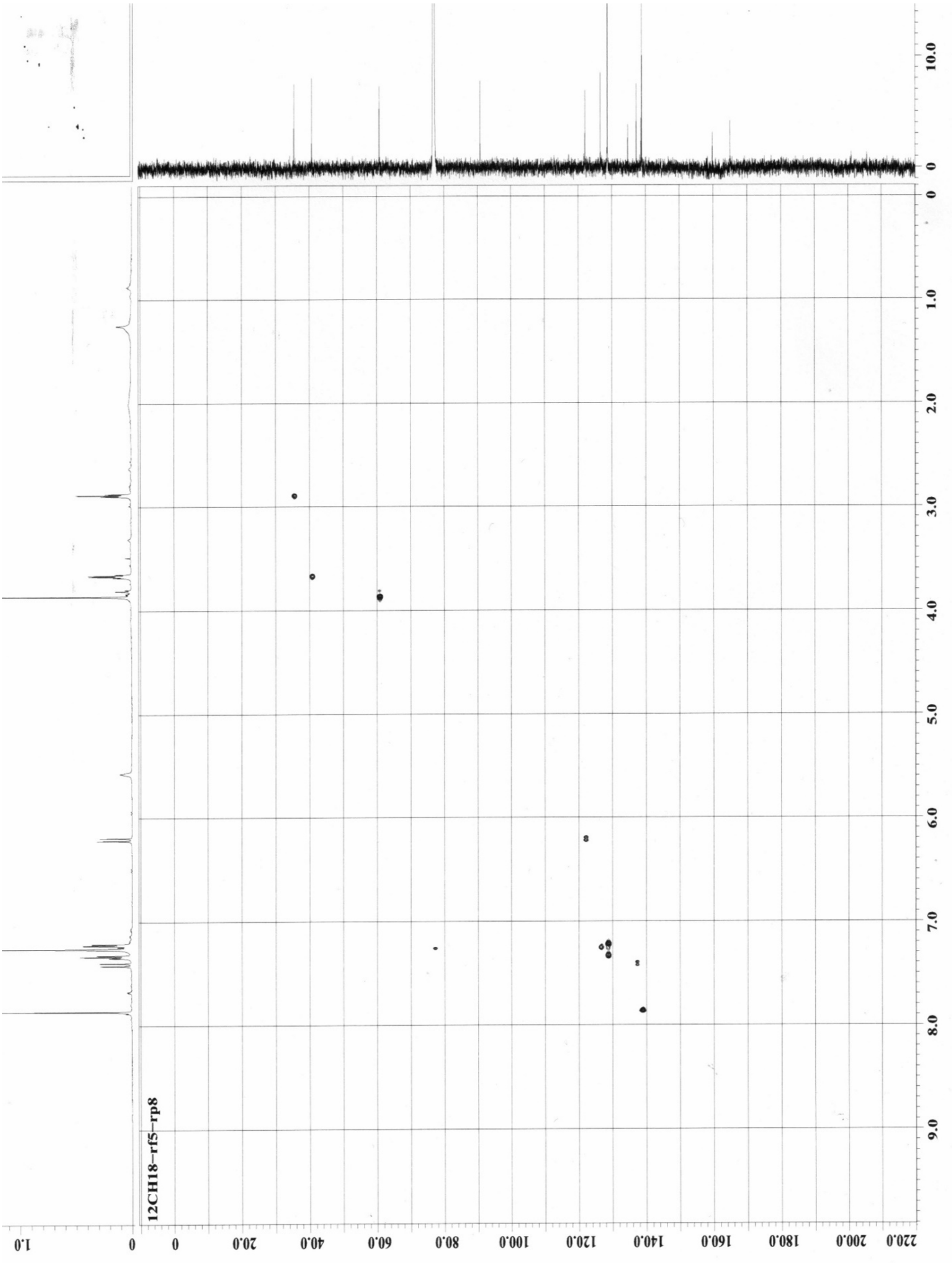

Figure S20. The *g*HSQC (600 MHz, $CDCl_3$) spectrum of apliamide D (**4**).

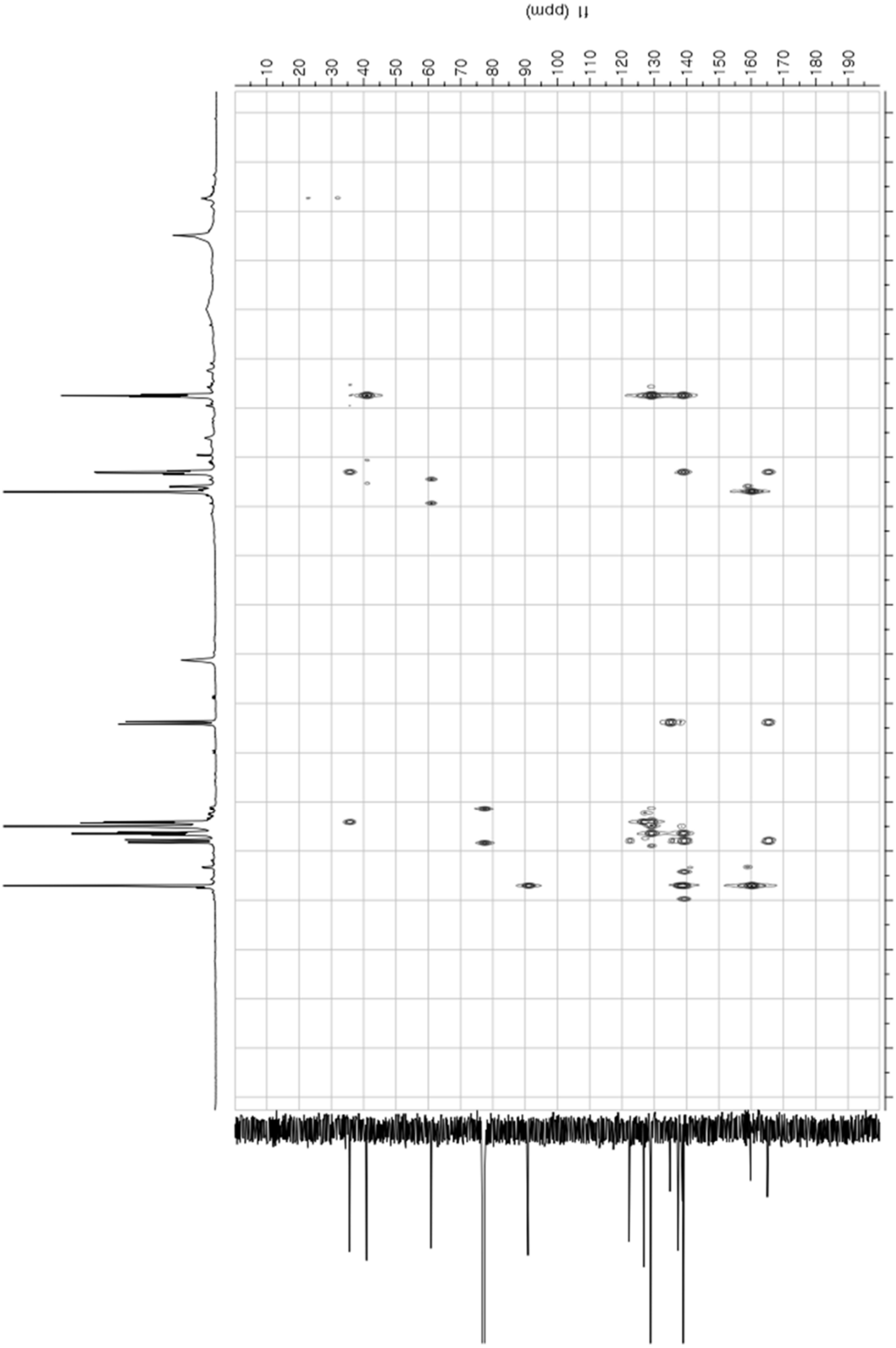

Figure S21. The *g*HMBC (600 MHz, $CDCl_3$) spectrum of apliamide D (**4**).

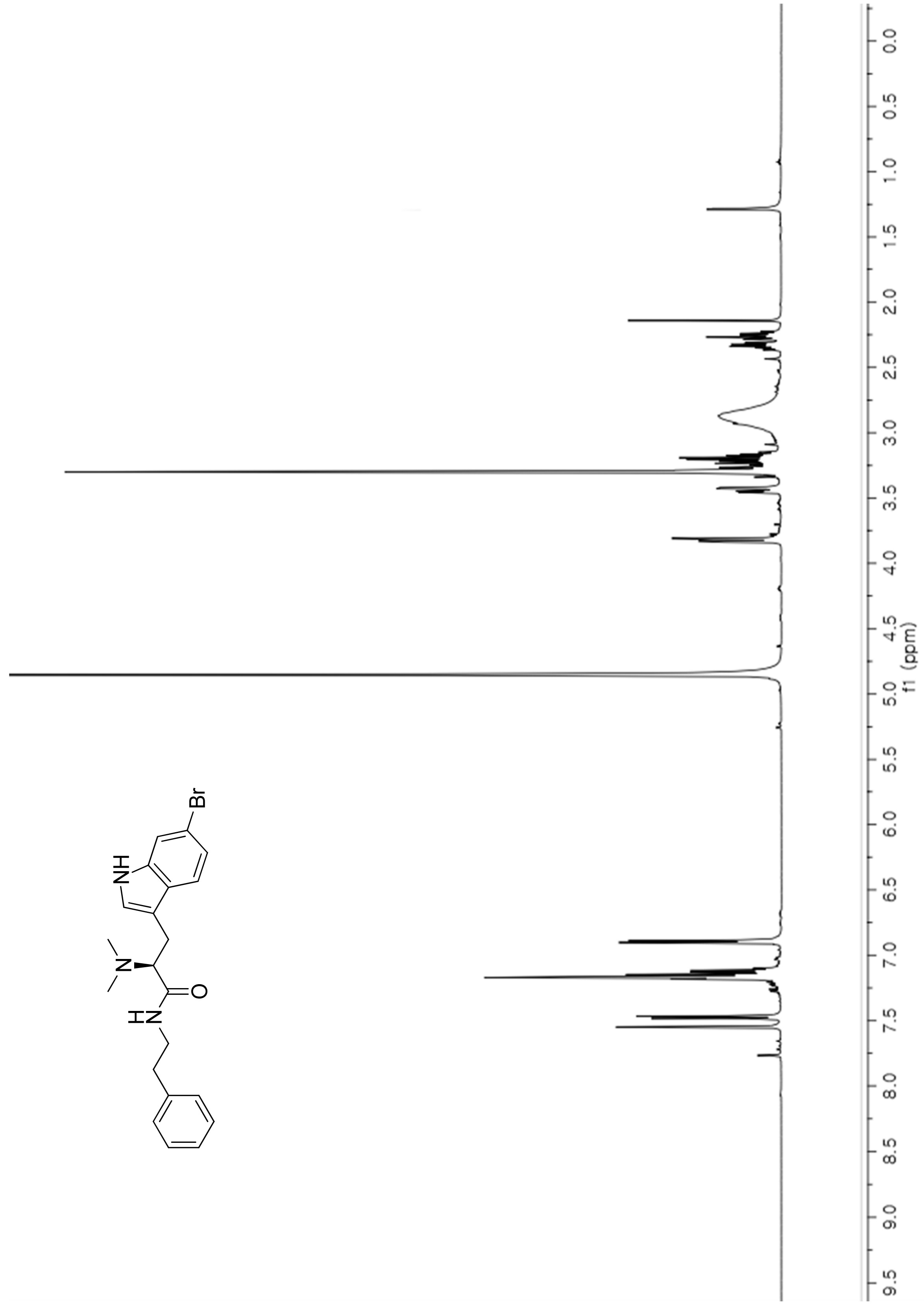

Figure S22. The ^{1}H NMR (600 MHz, MeOH-d_4) spectrum of apliamide E (**5**).

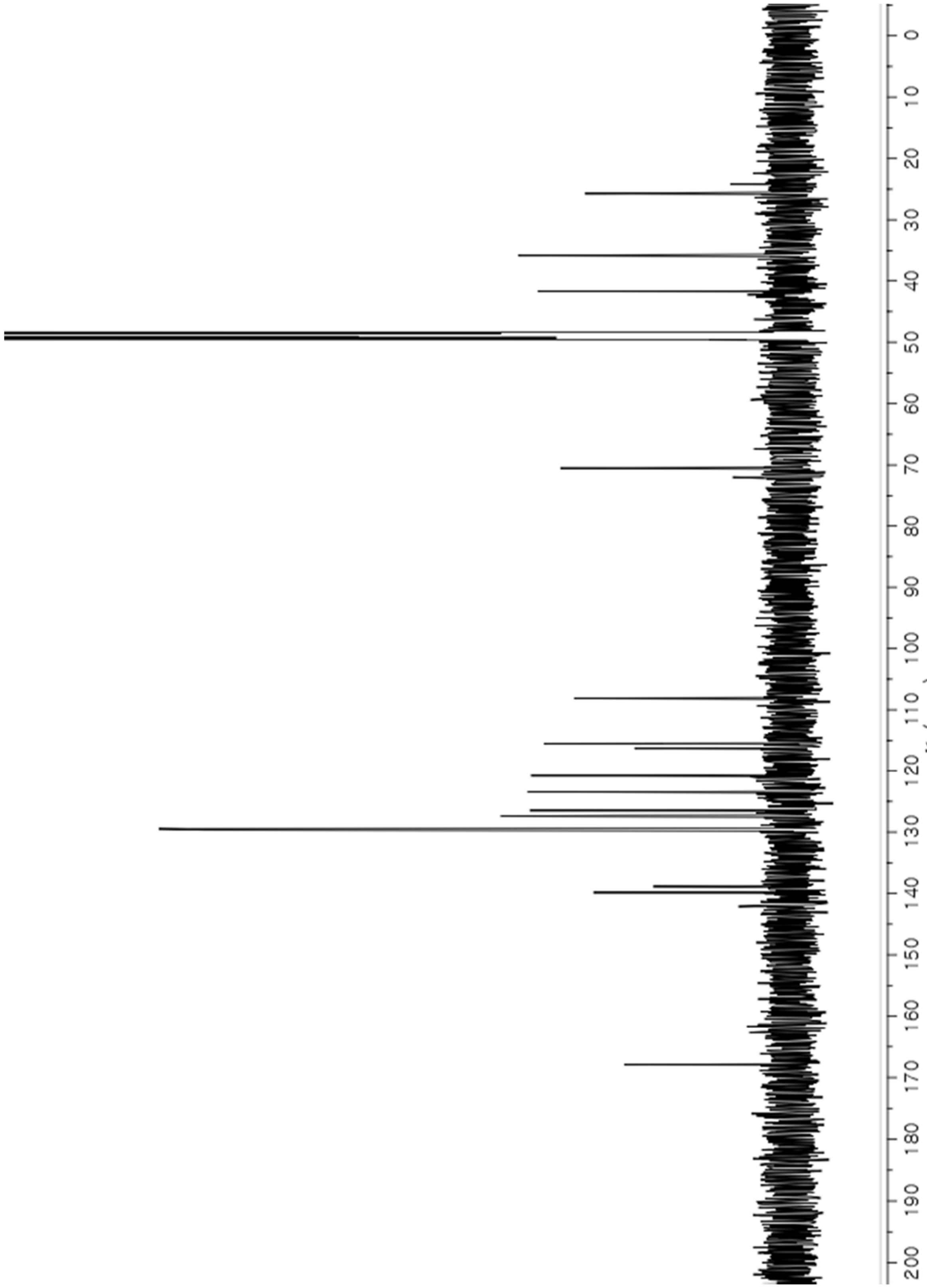

Figure S23. The ^{13}C NMR (150 MHz, MeOH-d_4) spectrum of apliamide E (**5**).

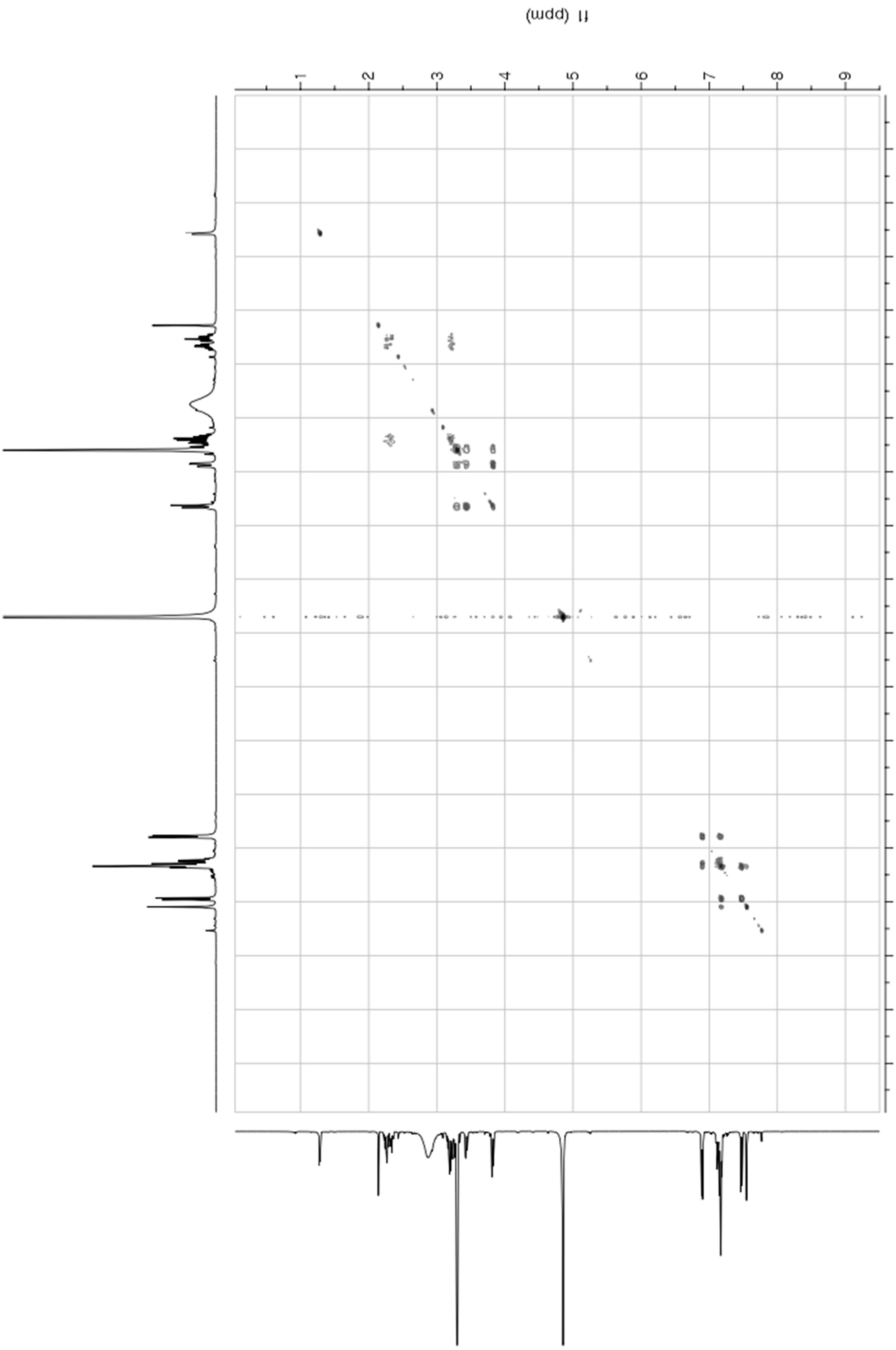

Figure S24. The COSY (600 MHz, MeOH-d_4) spectrum of apliamide E (**5**).

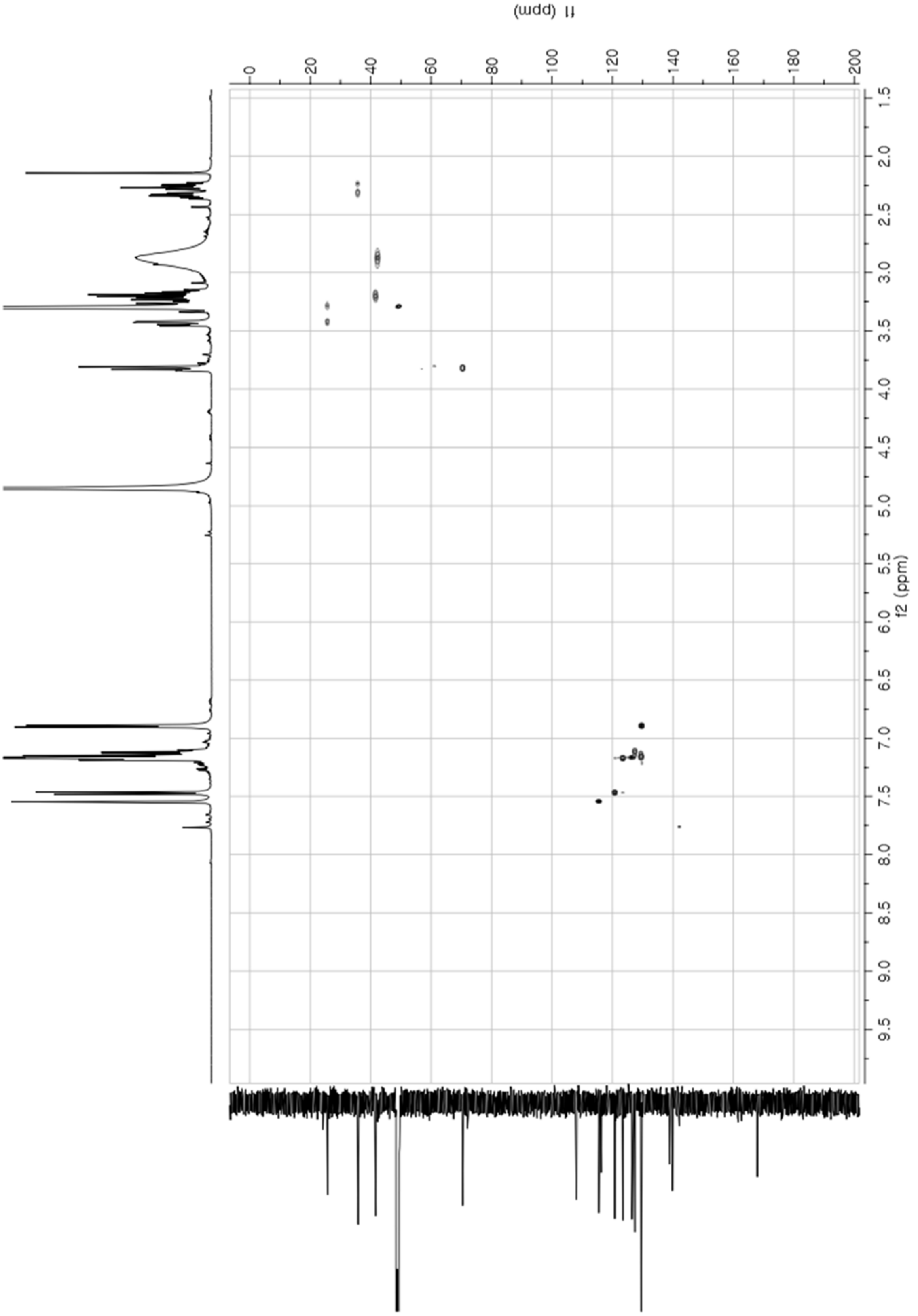

Figure S25. The *g*HSQC (600 MHz, MeOH-d_4) spectrum of apliamide E (**5**).

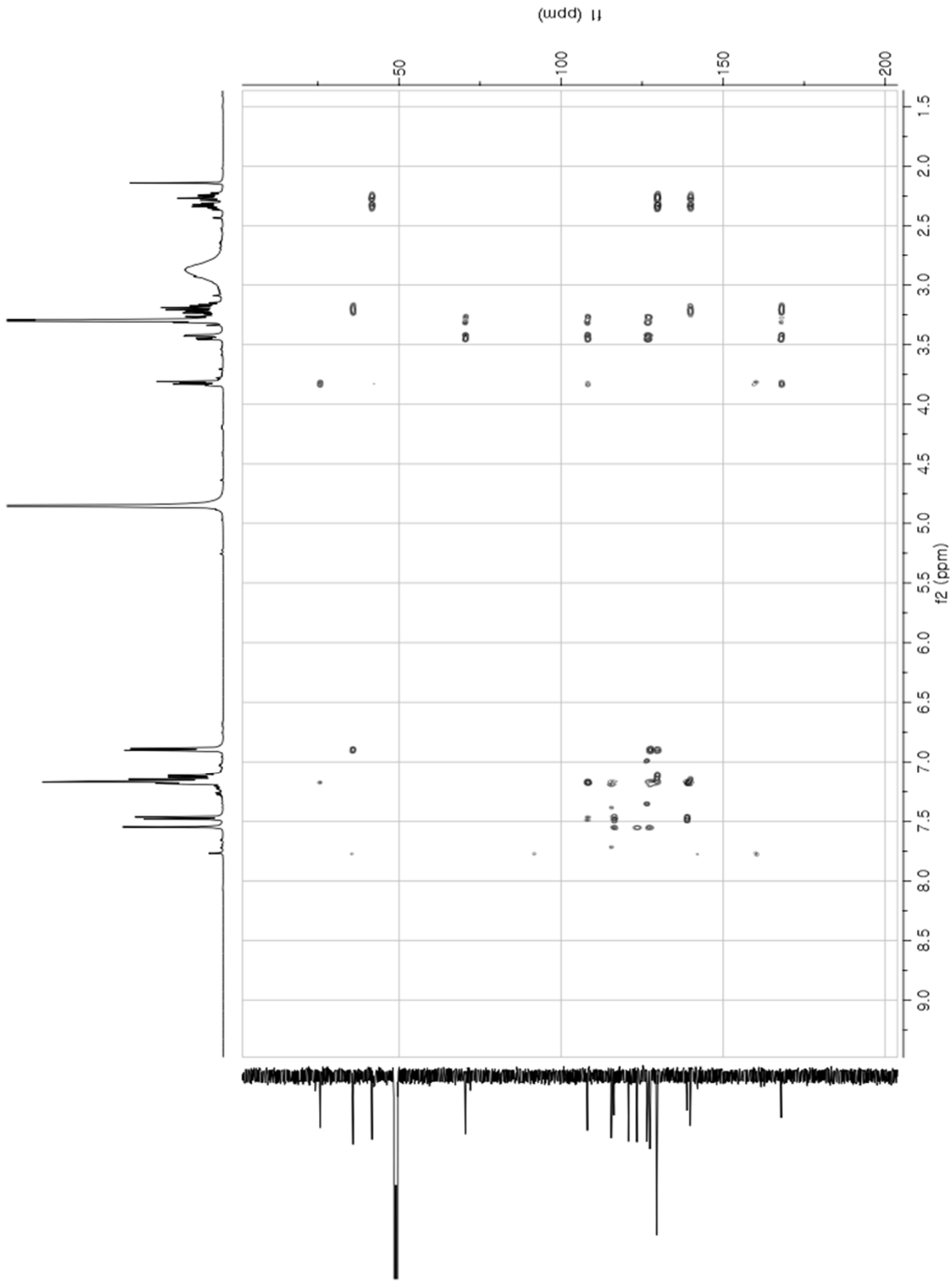

Figure S26. The *g*HMBC (600 MHz, MeOH-d_4) spectrum of apliamide E (**5**).

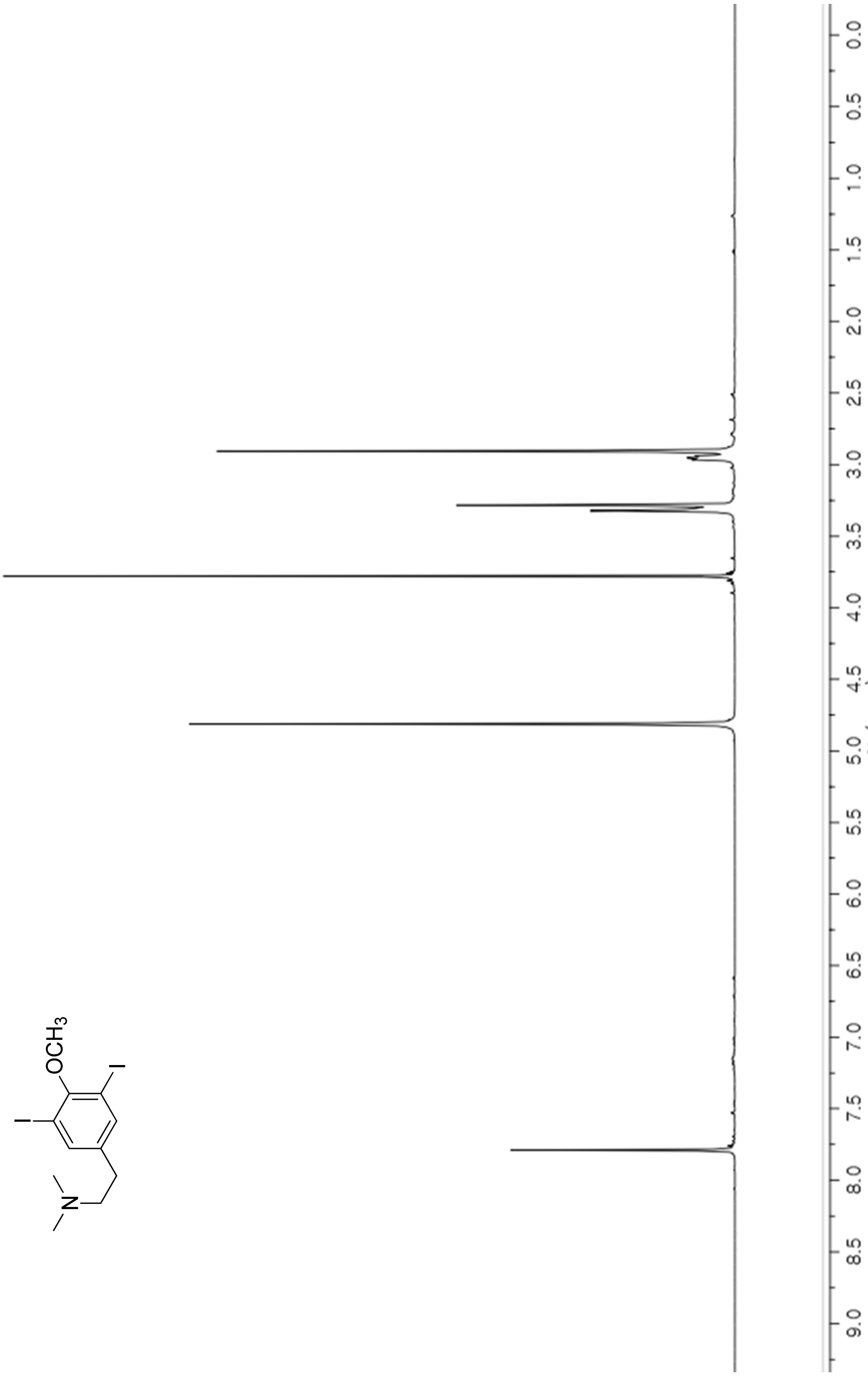

Figure S27. The ^{1}H NMR (600 MHz, MeOH-d_4) spectrum of apliamine A (**6**).

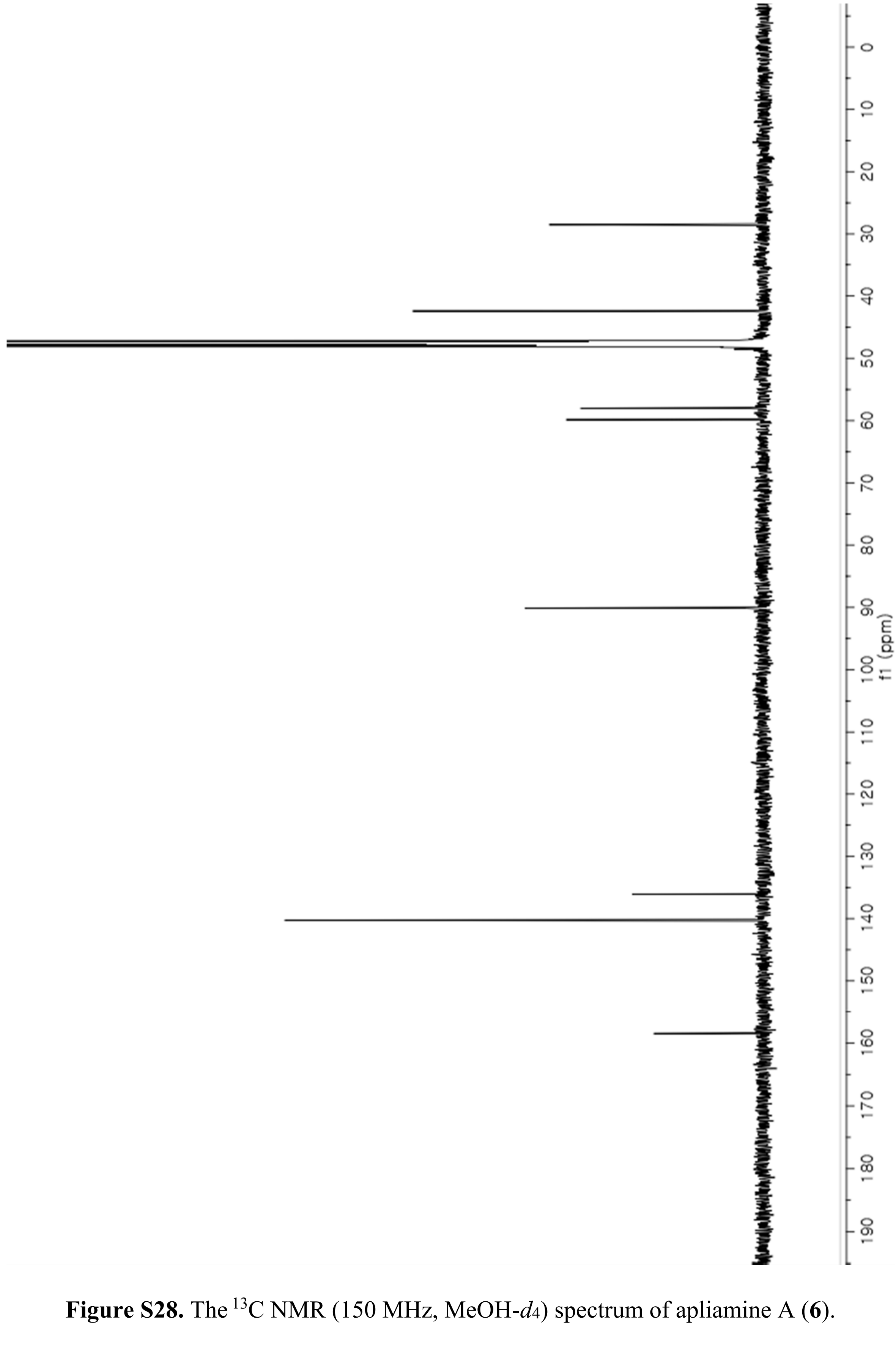

Figure S28. The ^{13}C NMR (150 MHz, MeOH-d_4) spectrum of apliamine A (**6**).

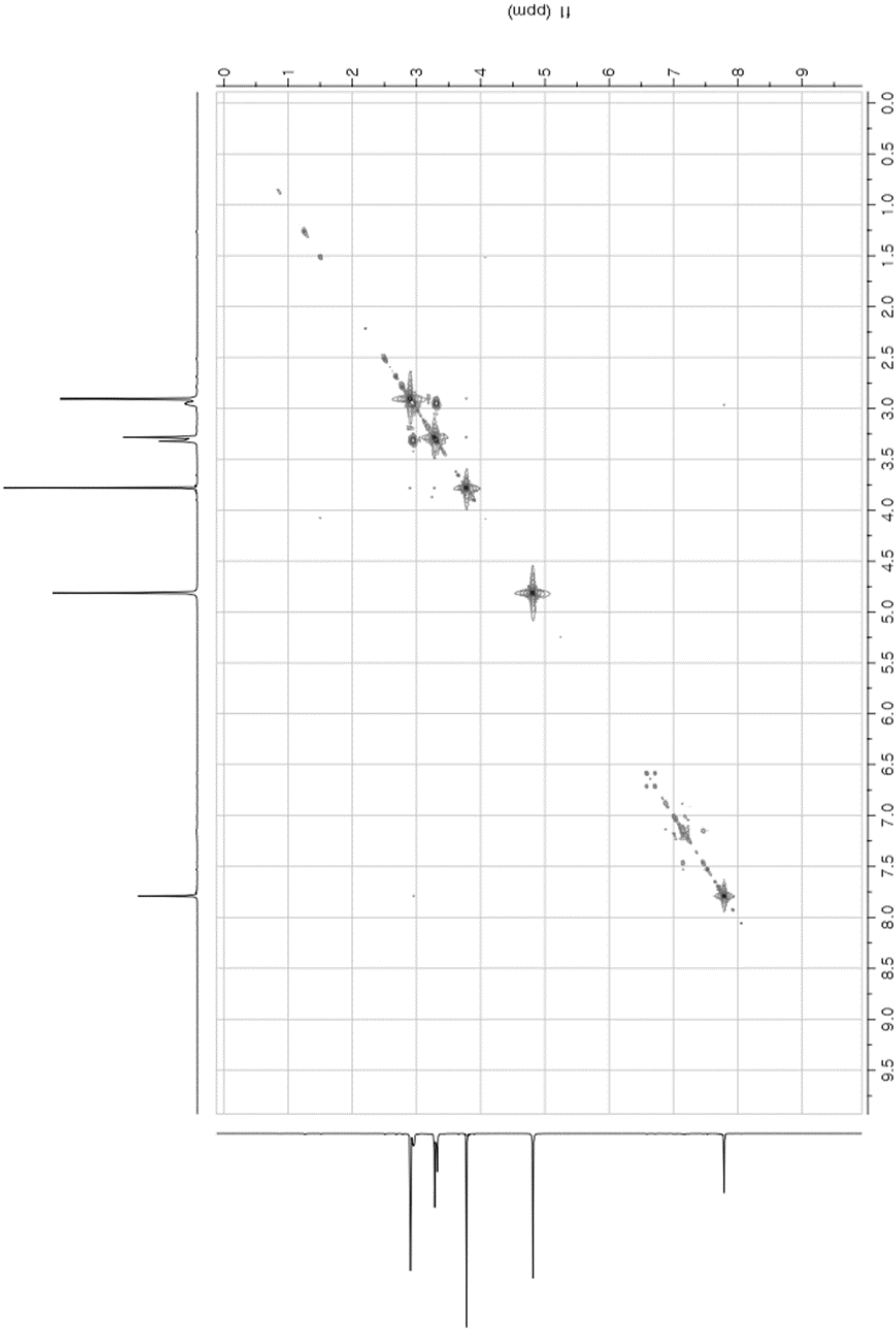

Figure S29. The COSY (600 MHz, MeOH-d_4) spectrum of apliamine A (**6**).

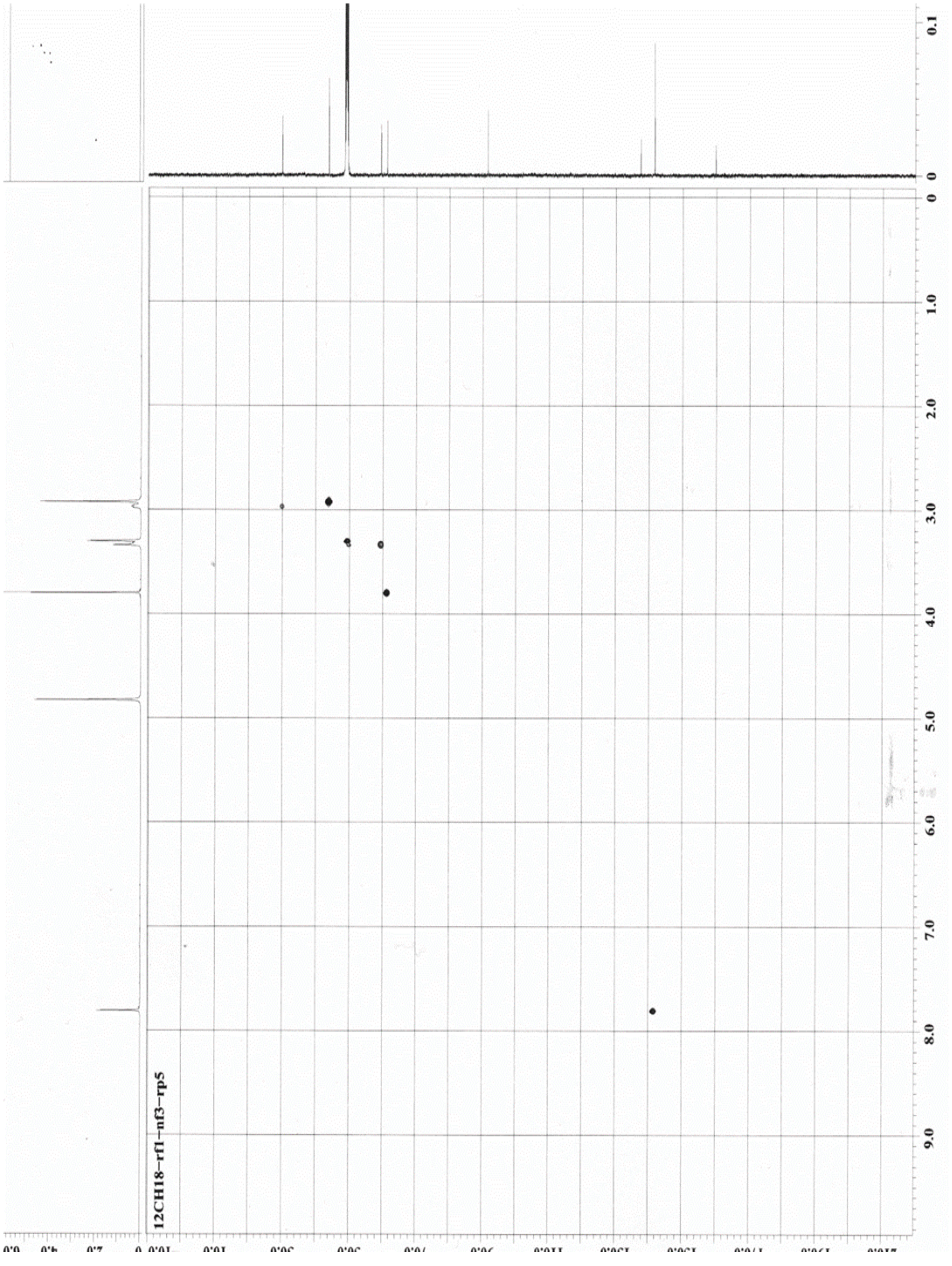

Figure S30. The *g*HSQC (600 MHz, MeOH-*d*4) spectrum of apliamine A (**6**).

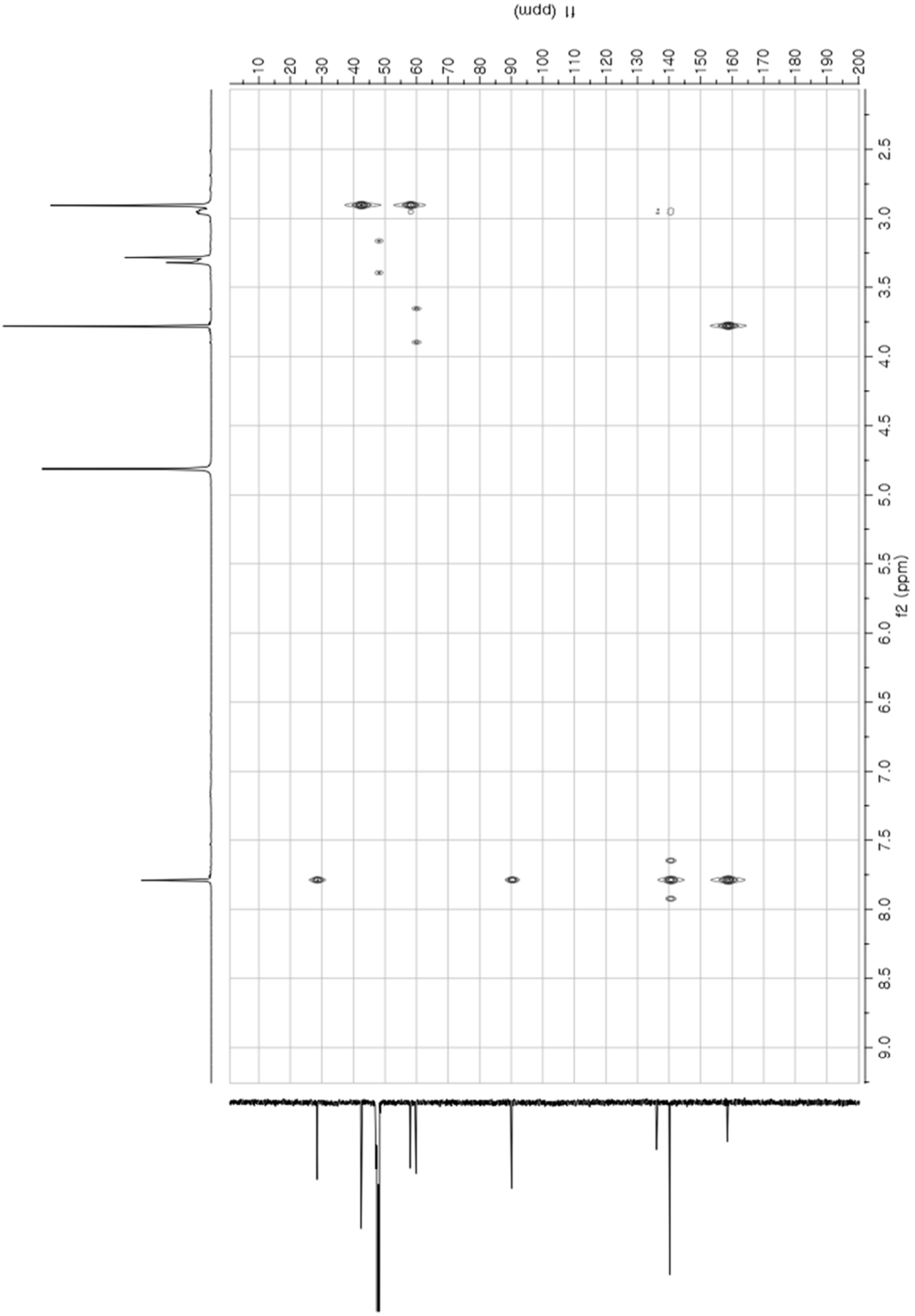

Figure S31. The *g*HMBC (600 MHz, MeOH-d_4) spectrum of apliamine A (**6**).

Table S1. The result of antibacterial and enzyme inhibition test [a].

	MIC (μg/mL)						IC_{50} (μM)	
	**Gram(+) bacterium**			**Gram(−) Bacterium**				
**Compound**	**A**	**B**	**C**	**D**	**E**	**F**	**Srt A**	**ICL**
**1**	>100	>100	>100	>100	>100	>100	>300	>100
**2**	>100	>100	>100	>100	>100	>100	>300	>100
**3**	>100	>100	>100	>100	>100	>100	>300	>100
**4**	>100	>100	>100	>100	>100	>100	>300	>100
**5**	>100	>100	>100	>100	>100	>100	>300	>100
**6**	>100	>100	>100	>100	>100	>100	>300	>100
Ampicillin	0.4	0.4	0.4	0.4	0.8	6.3		
*p*HMB [b]							109.2	
3-NP [c]								16.2

[a] A: *Staphylococcus aureus* (ATCC 6538p), B: *Bacillus subtilis* (ATCC 6633), C: *Micrococcus luteus* (IFO 12708), D: *Salmonella typhimurium* (ATCC 14028), E: *Proteus vulgaris* (ATCC 3851), F: *Escherichia coli* (ATCC 35270); [b] *para*-Hydroxymercuribenzoic acid; [c] 3-Nitropropionic acid.

© 2015 by the authors; licensee MDPI, Basel, Switzerland. This article is an open access article distributed under the terms and conditions of the Creative Commons Attribution license (http://creativecommons.org/licenses/by/4.0/).
